# The Lack of Alterations in Metabolites in the Medial Prefrontal Cortex and Amygdala, but Their Associations with Autistic Traits, Empathy, and Personality Traits in Adults with Autism Spectrum Disorder: A Preliminary Study

**DOI:** 10.1007/s10803-022-05778-7

**Published:** 2022-10-17

**Authors:** Yukihiko Shirayama, Kazuki Matsumoto, Fumio Osone, Akira Hara, Siqing Guan, Sayo Hamatani, Katsumasa Muneoka, Koichi Sato, Akihiro Okada, Tokuzou Yokokawa

**Affiliations:** 1 Department of Psychiatry, Teikyo University Chiba Medical Center, 3426-3 Anesaki, Ichihara, 299-0111 Japan; 2 Department of Radiology, Teikyo University Chiba Medical Center, Ichihara, Japan; 3 Department of Psychology, Sapporo International University, Sapporo, Japan

**Keywords:** Amygdala, Medial prefrontal cortex, Personality traits, Empathy, ASD, MRS

## Abstract

Proton magnetic resonance spectroscopy (^1^H-MRS) has shown inconsistent alterations in brain metabolites of adults with autism spectrum disorder (ASD). We investigated brain metabolites in the medial prefrontal cortex and amygdala of 24 drug-naive adults with ASD and no intellectual disability and 24 non-ASD control subjects, using 3 T ^1^H-MRS. Adults with ASD showed no significant differences from control in glutamate, glutamate plus glutamine, N-acetylaspartate, glycerophosphorylcholine plus phosphorylcholine, creatine plus phosphocreatine, or myo-inositol in either region. However, ASD subjects did show significant correlations of localized brain metabolites with autistic traits, empathy deficits, and personality traits using the Autism-Spectrum Quotient, Questionnaire of Cognitive and Affective Empathy, Interpersonal Reactivity Index, and NEO Personality Inventory-Revised. These findings should be taken as preliminary or exploratory.

**Supplementary Information:**

The online version contains supplementary material available at 10.1007/s10803-022-05778-7.

## Introduction

Autism spectrum disorder (ASD) is defined by deficits in social communication and social interaction (social-emotional reciprocity, nonverbal communicative behaviors, or developing and maintaining relationships), and restricted, repetitive patterns of behavior, interests or activities. It is suggested that those with autism have difficulties with emotional relationships due to deficits in empathy, which is hypothesized to have both cognitive and affective components. Cognitive empathy is the capacity to understand other people’s feelings, intentions, and beliefs on an intellectual level, while affective empathy is the emotional response to other people’s affective states or feelings (Davis, 1983; Singer, 2006). Adults with ASD and no intellectual disability showed impairments of cognitive empathy in scores for perspective taking on the Interpersonal Reactivity Index (IRI) (Lombardo et al., 2007; Rogers et al., 2007). In our recent study, adults with ASD and no intellectual disability showed impairments of cognitive empathy rather than affective empathy on the IRI and Questionnaire of Cognitive and Affective Empathy (QCAE), and personality traits on the NEO Personality Inventory-Revised (NEO), and multiple regression analysis demonstrated that perspective taking on the QCAE and extraversion on the NEO were good predictor variables to autistic traits on the Autism-spectrum quotient (AQ) (Shirayama et al., 2022).

The neural basis of empathy is reported to involve the anterior cingulate and insula (Bernhardt & Singer, 2012). Cognitive empathy is associated with the medial prefrontal cortex and cingulate cortex, whereas affective empathy is associated with the insula (Eres et al., 2015; Fan et al., 2011). Cognitive empathy may partially involve the mechanisms underlying the theory of mind, which is shown to be associated with the medial prefrontal cortex and temporoparietal junction (Schurz et al., 2014). The medial prefrontal cortex is activated in the context of mentalizing or theory of mind tasks when people are attending to certain states of the self or others (Frith and Frith,  1999; Jackson et al., 2006), and the anterior cingulate cortex plays an important role in the executive control of attention (Fan et al., 2005).

Cognitive empathy deficits in ASD could involve impairments in social cognition, in which the amygdala might be implicated (Blair et al., 2008). The amygdala is required to recognize emotions, social interactions, and evaluating the social value of a stimulus (Adolphs, 2001). The social brain's neural network includes the amygdala, suggesting the amygdala theory of autism (Baron-Cohen et al., 2000; Nacewicz et al., 2006; Schulkin, 2007). The developmental dysfunction of the orbitofrontal-amygdala circuit is critical for the deficits in self-regulation of social-emotional behavior in autism (Bachevalier & Loveland, 2006). When emotional face processing was needed, the amygdala showed reduced activation in autistic subjects (Pierce et al., 2001). In another study, adult individuals with high-functioning ASD, including Asperger syndrome, showed less activation of the amygdala and frontal cortex when processing facial expressions (Critchley et al., 2000).

A study of the default mode network of ASD subjects found that the strength of the resting-state functional connectivity came from the core region of the medial prefrontal cortex in ASD participants (Jung et al., 2014). Social cognitive dysfunction such as self-referential processing and theory of mind involved the medial prefrontal cortex functional connectivity in an adult group of ASD (reviewed by Padmanabhan et al., 2017). In a PET study, subjects with ASD showed glucose hypometabolism in the anterior cingulate cortex, but not in the amygdala (Haznedar et al., 2000). Functional MRI studies reported enhanced amygdala activation in ASD subjects (Dalton et al., 2005; Kleinhans et al., 2009a), and that amygdala was involved in interpersonal space permeability and flexibility in ASD adults (Massaccesi et al., 2021). Whereas, individuals with ASD showed diminished activity in the medial prefrontal cortex and insula for conflicting nonverbal information (Watanabe et al., 2012), and activation in the medial prefrontal cortex during thresholded unsigned prediction errors (Mosner et al., 2019). Interestingly, the medal prefrontal cortex was activated in individuals with ASD in response to autistic characters and in typically developing individuals in response to non-autistic characters (Komeda et al., 2015). Furthermore, oxytocin increased the originally diminished brain activity in the medial prefrontal cortex in ASD (Watanabe et al.,  2014). These areas predicted autistic communication deficits in ASD. An MRI study showed a decreased-volume medial prefrontal cortex was shown in high-functioning ASD subjects compared to normal controls (Haznedar et al., 2000). However, studies of amygdala volumes in adults with ASD have shown conflicting results: reduced amygdala volume in some (Nacewicz et al., 2006; Pierce et al., 2001) and increased volume in others (Abell et al., 1999; Howard et al., 2000), with one study showing no difference (Haznedar et al., 2000). It is of note that the gray matter volume of the amygdala was correlated positively with cognitive and affective empathy, but negatively with cognitive alexithymia (Goerlich-Dobre et al., 2015).

Glutamatergic signaling, particularly as it relates to synaptic plasticity, is believed to be involved in the development of ASD (Veenstra-VanderWeele & Blakely, 2012). Previous studies utilizing 3 T Proton magnetic resonance spectroscopy (^1^H-MRS) demonstrated that adults with ASD had a decreased glutamate in the anterior cingulate cortex (Tebartz van Elst et al., 2014) or no changes in glutamate in the medial prefrontal cortex (Horder et al., 2018). Meanwhile, three studies have reported no differences in glutamate plus glutamine (Glx) in the medial prefrontal cortex of ASD patients compared to controls (Aoki et al., 2012; Endres et al., 2017; Horder et al., 2018), whereas two studies showed decreased levels of Glx in the medial prefrontal cortex of ASD subjects (Bernardi et al., 2011; Tebartz van Elst et al., 2014). Recently, cannabidiol increased Glx in the medial prefrontal cortex, but decreased GABA in the adults with ASD using MRS (Pretzsch et al., 2019). Neuronal network hyperexcitability in cerebral cortex autism was supposed glutamate and GABA through maternal metabolic morbidity (Rivell & Mattson, 2019).

Adults with ASD showed increased N-acetylaspartate (NAA) levels in the medial prefrontal cortex (Aoki et al., 2012; Murphy et al., 2002), or increased NAA/choline ratio in the anterior cingulate cortex (Oner et al., 2007), whereas other studies showed significantly decreased NAA signals in the pregenual anterior cingulate of ASD adults (Tebartz van Elst et al., 2014), or a reduction in the NAA/creatine ratio in the anterior cingulate cortex of ASD adults (Libero et al., 2015), and no changes in NAA levels in the prefrontal cortex of ASD (Endres et al., 2017). NAA levels are interpreted as a marker of neuronal density and/or mitochondrial function (Clark, 1998). On the other hand, there was one report of no abnormalities in NAA levels in the amygdala of high-functioning ASD and Asperger syndrome (Kleinhans et al., 2009b).

Choline-containing compounds, glycerophosphorylcholine plus phosphorylcholine (GPC + PC), represent membrane turnover. GPC is one of the breakdown products of phospholipids, whereas PC is a precursor of the phospholipid membrane (Shirayama et al., 2004). High levels of GPC + PC were also reported in the medial prefrontal cortex of Asperger syndrome (Murphy et al., 2002). However, there were no significant changes in Cho (GPC + PC) in the amygdala of high functioning ASD and Asperger syndrome (Kleinhans et al., 2009b).

High concentrations of creatine plus phosphocreatine (Cr + PCr) were reported in the medial prefrontal cortex of Asperger syndrome (Murphy et al., 2002). On the contrary, no significant difference in Cr + PCr was shown in the amygdala of high functioning ASD and Asperger syndrome compared to controls (Kleinhans et al., 2009b).

Myo-inositol is a putative marker of glial cells because myo-inositol is transiently but actively transported into astrocytes (Barres, 2008). Reduced levels of myo-inositol were shown in the anterior cingulate of adults with high-functioning ASD compared to controls (Endres et al., 2017), whereas three studies showed no significant differences in myo-inositol levels in the medial frontal cortex of ASD (Aoki et al., 2012; Bernardi et al., 2011; Tebartz van Elst et al., 2014). On the other hand, one study showed no significant difference in myo-inositol in the amygdala of ASD adults (Kleinhans et al., 2009b).

Increases in magnetic field strengths make it possible to acquire proton (^1^H) spectra from a smaller volume of interest (VOI) with fewer scan numbers than before (Shirayama et al., 2010). Recently, reliable results from the amygdala can now be obtained using new techniques with saturation bands for shimming (Nacewicz et al., 2012). Utilizing 3 T ^1^H-MRS and saturation bands, we obtained acceptable measures of glutamate from the amygdala and the medial prefrontal cortex using a volume of 8 cm^3^ (Shirayama et al., 2017).

This study aimed to investigate the levels of NAA, glutamate, Glx, Cr + PCr, GPC + PC, myo-inositol, and glutamine in the medial prefrontal cortex and amygdala of adults with ASD and no intellectual disability using 3 T ^1^H-MRS and saturation bands. Next, we examined the relationship between brain metabolites, and autistic traits on the AQ, cognitive and affective empathy scores on the QCAE and IRI, and personality traits on the NEO.

## Methods

### Subjects

The participants consisted of 24 adults with ASD and 24 healthy control subjects. The inclusion criterion required patients to be drug-naïve. Participating adults with ASD were recruited from the outpatient clinic of Teikyo University Chiba Medical Center, and met the DSM-V criteria for autism spectrum disorders (American Psychiatric Association, 2013), requiring consensus based on more than 4 months of longitudinal follow-up examination by trained psychiatrists and licensed clinical psychologists. The adults with ASD had no other psychiatric disorders at enrollment, including depression. The subjects with ASD did not have a history of delay in language development. Based on the full IQ, the patients with ASD included in our study were high functioning. Other criteria for exclusion were a history of head trauma, seizures or other neurological disorders, mental retardation, alcohol, and substance abuse. This research was approved by the ethics committee of Teikyo University School of Medicine (ethical committee approval No. 17–105) and was performed following the Declaration of Helsinki. Written informed consent was obtained after the procedures had been fully explained to each participant.

### Demographics

Participants in this study were almost the same as those in our previous study (Shirayama et al., 2022), where we have already presented and discussed these data. Table 1 summarizes the demographics of participants in this study. The ASD adults with no intellectual disability showed significant higher scores for autistic traits on the AQ. The ASD adults showed significant differences from non-ASD controls in their scores for perspective taking, online simulation, and peripheral responsivity on the QCAE. Distinctly, the ASD cohorts also showed significantly lower scores for cognitive empathy but not affective empathy on the QCAE. The ASD adults showed significant deficits in perspective taking and empathic concern scores on the IRI. Adults with ASD showed significantly higher scores for neuroticism and lower scores for extraversion and conscientiousness on the NEO compared with non-ASD controls. The depressive level on the BDI was significantly higher in ASD adults compared with non-ASD controls. Full IQ showed significant differences between ASD adults and non-ASD controls.

**Table 1:** Participant demographics

	Non-ASD control (n = 24)	ASD (n = 24)	*p*-values	*t*-values (df)	Effect size
Age, years (range)	30.0 ± 6.4 (23–44)	27.5 ± 7.5 (18–44)	0.213	1.24 (46)	0.358
Sex (male/female)	9/15	14/ 10	0.163		
AQ	17.2 ± 6.4	32.7 ± 6.5	< 0.001 ***	8.32 (46)	2.403
ADOS-2 for clinical use	0.5 ± 1.7	7.1 ± 2.7	< 0.001 ***	10.18 (46)	2.925
BDI	5.3 ± 6.3	10.0 ± 6.5	0.014 *	2.54 (46)	0.734
Full IQ	108.0 ± 13.1	97.2 ± 11.7	0.004 **	3.01 (46)	0.869
Verbal IQ	107.6 ± 12.9	98.4 ± 13.3	0.020 *	2.41 (46)	0.697
Performance IQ	107.3 ± 13.0	96.5 ± 13.8	0.008 **	2.79 (46)	0.805
< QCAE >					
Perspective-taking	34.6 ± 6.8	24.3 ± 7.7	< 0.001 ***	4.91 (46)	1.417
Online simulation	33.5 ± 5.2	26.5 ± 7.6	< 0.001 ***	3.72 (46)	1.075
Emotion contagion	13.3 ± 2.8	13.2 ± 4.3	0.905	0.09 (46)	0.027
Proximal responsivity	11.9 ± 2.7	10.7 ± 3.3	0.147	1.37 (46)	0.398
Peripheral responsivity	14.2 ± 2.5	11.9 ± 3.2	0.008 **	2.77 (46)	0.801
Cognitive empathy	68.1 ± 10.8	50.6 ± 13.4	< 0.001 ***	4.98 (46)	1.438
Affective empathy	39.5 ± 6.5	35.7 ± 8.0	0.082	1.80 (46)	0.521
< IRI >					
Perspective taking	20.9 ± 2.9	16.6 ± 3.6	< 0.001 ***	4.55 (46)	1.315
Empathic concern	20.3 ± 2.9	18.7 ± 2.4	0.045 *	2.08 (46)	0.601
Personal distress	17.5 ± 4.3	19.3 ± 4.6	0.178	1.40 (46)	0.404
Fantasy	19.9 ± 3.7	17.7 ± 4.4	0.061	1.87 (46)	0.541
< NEO >					
Neuroticism	55.4 ± 11.1	67.8 ± 10.7	< 0.001 ***	3.94 (36)	1.137
Extraversion	50.8 ± 12.3	34.7 ± 8.7	< 0.001 ***	5.23 (46)	1.511
Openness	51.8 ± 6.7	49.4 ± 9.4	0.296	1.02 (46)	0.294
Agreeableness	46.5 ± 11.5	40.6 ± 12.4	0.096	1.70 (46)	0.493
Conscientiousness	44.8 ± 10.8	36.6 ± 11.4	0.015 **	2.55 (46)	0.738

Data are mean ± SD. *p < 0.05, **p < 0.01, ***p < 0.001 compared to non-ASD controls (Student’s t-test). Effect size represents a sample-based estimate of the quality. *AQ* autism spectrum quotient; *ADOS*-2 autism diagnostic observation schedule, 2nd edition; *BDI* beck depression inventory; *IQ* intelligence quotient; *IRI* interpersonal reactivity index; NEO, NEO Personality Inventory-Revised;* QCAE* questionnaire of cognitive and affective empathy

### Psychological Tests for Autistic Traits, Empathy and Personality Traits

The autism-spectrum quotient (AQ) was used to assess autistic traits (Baron-Cohen et al., 2001), covering five areas: social skills, attention switching, attention to detail, communication, and imagination.

The Questionnaire of Cognitive and Affective Empathy (QCAE) was used to assess subjects’ levels of cognitive and affective empathy (Reniers et al., 2011). The cognitive subcomponents are perspective taking and online simulation, and the affective empathy subcomponents are emotional contagion, proximal responsivity, and peripheral responsivity.

The Interpersonal Reactivity Index (IRI) was used to assess dispositional empathy, consisting of four subscales: perspective taking, fantasy, empathic concern, and personal distress (Davis, 1983; Rankin et al., 2005). The perspective taking scale assesses one’s ability to arrive at cognitive understanding of what another person thinks or feels.

Personality traits were assessed using NEO Personality Inventory-Revised (NEO). NEO utilized the five-factor model of personality: neuroticism, extraversion, openness, agreeableness, and consciousness (Costa & McCrae, 1997).

Intelligence quotient (IQ), including full scale IQ, verbal IQ, and performance IQ, were estimated using the Wechsler Adult Intelligence Scale, 3rd edition (WAIS-III) (Wechsler, 2002). Inclusion IQ criteria were > 80.

The Beck Depression Inventory score (BDI) was used to assess depressiveness (Beck et al., 1961).

### ^1^H-MRS Methods

**Fig. 1:**
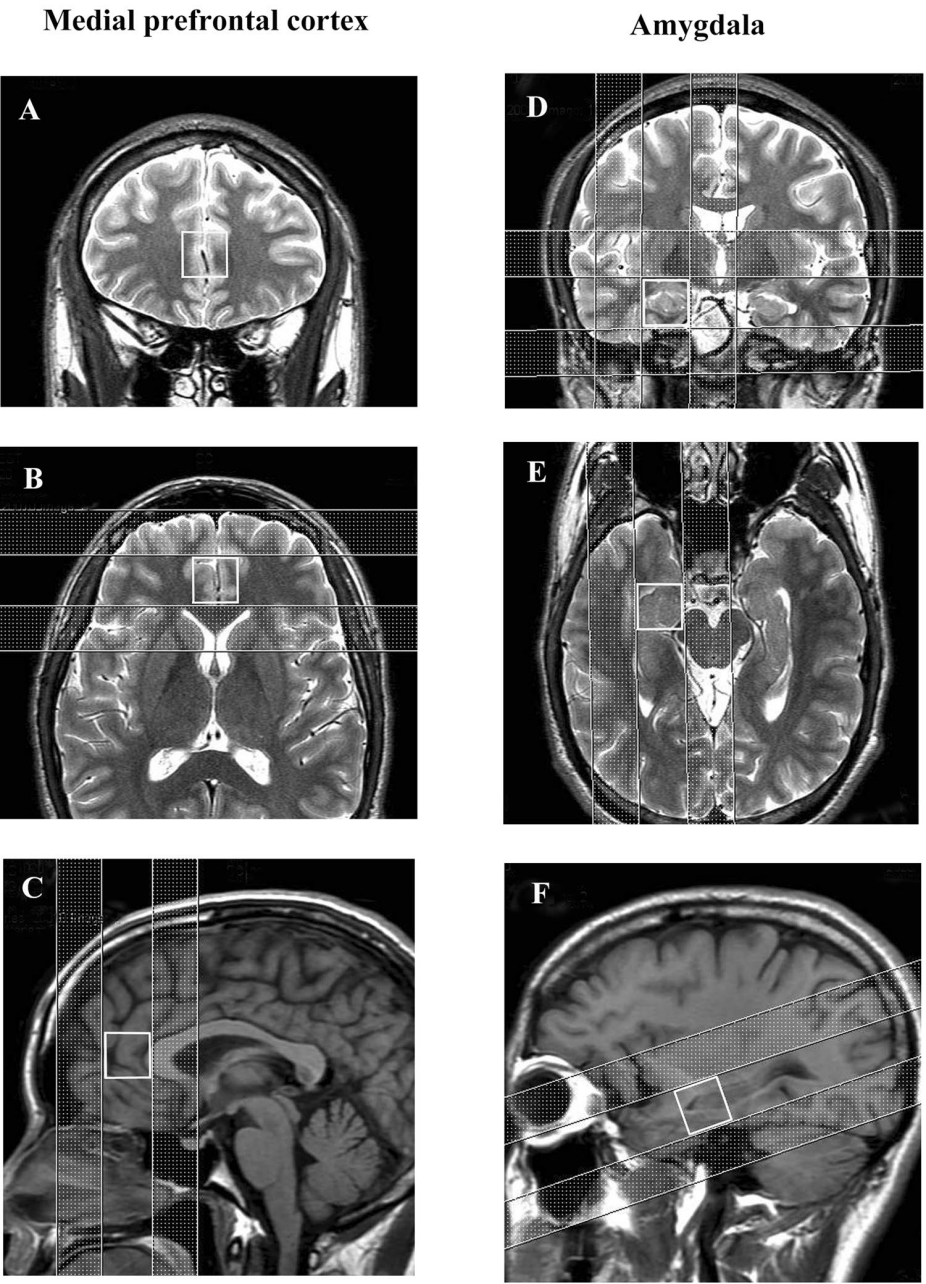
Voxels of interest in medial prefrontal cortex **A**, **B**, **C**, and amygdala **D**, **E**, **F** in coronal, axial and sagittal sections, respectively. Coronal and axial views were chosen under T2-weighted image pulses. Sagittal views were chosen under T1-weighted image pulses. Saturation bands were placed for the outer volume

^1^H-MRS data was acquired from all subjects using a Discovery MR750 (GE, Milwaukee, WI, USA), operated at 3 T. The ^1^H nuclear magnetic resonance used a 12-channel head coil. The volume of interest (VOI) location was chosen under the guidance of T2-weighed image pulses (repetition time 5 s, echo time 98 ms, slice thickness 3 mm) and T1-weighed image pulses (repetition time 2 s, echo time 25 ms, slice thickness 5 mm) for the cubic voxel to include the medial prefrontal cortex (20 × 20 × 20 mm), and right amygdala (20 × 20 × 20 mm) (Fig. 1). The VOI for the medial prefrontal cortex mainly includes the anterior cingulate cortices. The mean percentage of the MRS voxels filled with amygdala was 90%.

Saturation bands for the outer volume were placed to reduce signals external to the VOI for an improved signal-to-noise ratio (Fig. 1). We set 2 saturation bands along the anterior–posterior (AP) border of the VOI for the medial prefrontal cortex. We set 4 saturation bands along the superior-inferior (SI) border and the right-left (RL) border of the VOI for the amygdala (Shirayama et al., 2017).

Then, regional auto-shimming was performed to afford the appropriate excitation volume. We shimmed to a prescan line width (full width at half maximum, FWHM) less than 9 Hz and water suppression (WS) above 99% in the medial prefrontal cortex, and line width less than 12 Hz and WS above 98 in the amygdala, respectively. ^1^H spectra were acquired using a point-resolved spectroscopy sequence (PRESS) (repetition time 4 s, echo time 30 ms, with 128 and 256 scans in the medial prefrontal cortex and amygdala, respectively). The mean signal-to-noise ratios (SNR) were 13.5 and 20.0 in the medial prefrontal cortex and amygdala, respectively. No statistically significant differences were found in the FWHM and SNR between the ASD patients and controls in the two regions (Supplementary Table S1). The total examination time was almost 65 min.

**Fig. 2.**
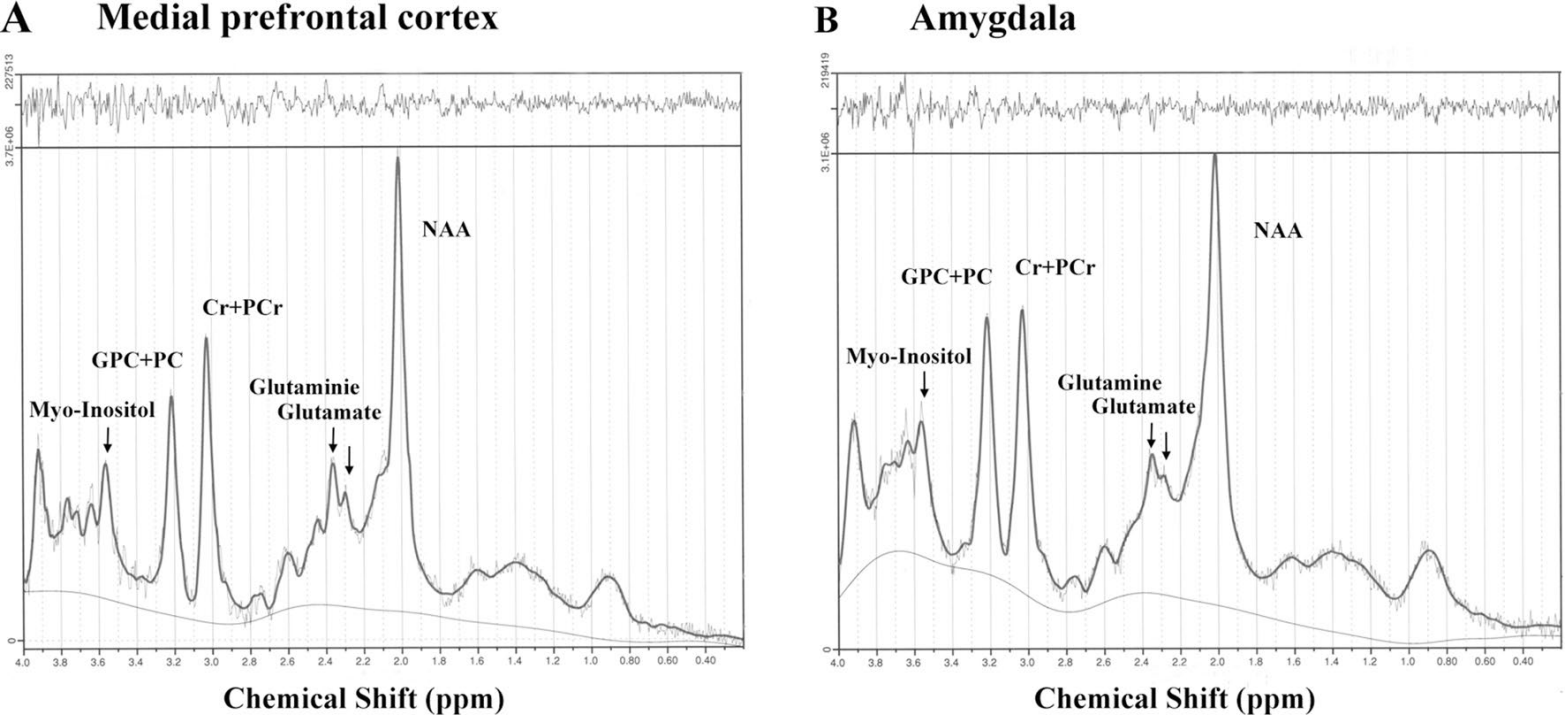
^1^H MRS data from the medial prefrontal cortex and amygdala. Spectra of the unfiltered data are superimposed with the LCModel fit **A**. Residual noise **B.** Cr + PCr, creatine plus phosphocreatine; GPC + PC, glycerophosphorylcholine plus phosphorylcholine; NAA, N-acetyl-L-aspartate; ppm, parts per million.

Spectra were analyzed using the linear combination model (LCModel version 6.3 1-H) (Provencher, 2001; Shirayama et al., 2017) (Fig. 2). Metabolite concentrations for glutamate, Glx, NAA, GPC + PC, Cr + PCr, and myo-inositol were estimated. The standard GE libraries of model metabolite spectra provided with the LCModel were used as a basis reference for time domain fitting. The reliability of metabolite quantification was judged by the standard deviation of the fits, which was expressed as the percentage standard deviation (%SD) of the estimated concentration by Cramer-Rao lower bounds (CRLB) in the LCModel. For metabolites, our criterion for the reliability of the spectral fit was less than 10%SD. The mean CRLB values in the medial prefrontal cortex were 7.4 for glutamate, 6.6 for Glx, 4.2 for NAA, 4.2 for GPC + PC, 3.7 for Cr + PCr, and 6.2 for myo-inositol. The mean CRLB values in the amygdala were 7.6 for glutamate, 7.8 for Glx, 3.8 for NAA, 3.5 for GPC + PC, 3.5 for Cr + PCr, and 4.7 for myo-inositol. For glutamine, spectra with > 25% SD were excluded, resulting in a loss of one to four data points for each group. The mean CRLB values for glutamine were 18.0 in the medial frontal cortex and 17.3 in the amygdala. Since macromolecular resonances are prominent under PRESS with an echo time of 30 ms, levels of macromolecules (MMs) such as MM09 and MM20 were determined.

Segmentation was performed on the VOI, T2–weighted images using the equipped software (GE Healthcare Advantage Workstation, version 4.6), tracing and calculating the region of interest. The distribution of tissue and cerebrospinal fluid (CSF) was determined for voxel tissue composition. The mean voxel compositions of CSF were 6.5% in the medial prefrontal cortex, and 4.5% in the amygdala. No statistically significant differences were found in CSF proportions between the ASD patients and controls in the two regions (Supplementary Table S3). Metabolites values were corrected based on CSF volumes using the following formula: Cc = C/(1-Fcsf), where C is the original concentration, Cc is the corrected measure, and Fcsf is the portion of CSF volume in the voxel.

### Statistical Analysis

The chi-square test was used for categorical variables. Independent *t-*tests were used to determine differences between groups. The effect size was calculated using G-power (3.1). Coefficients of brain metabolites with psychological test scores were estimated by Pearson coefficients. Normality was checked by Kolmogorov–Smirnov test and Shapiro–Wilk test. Additional regression analysis with stepwise method were conducted, treating BDI, full IQ, verbal IQ and performance IQ as covariates. Nonparametric Spearman's rank correlation coefficients were exerted as an additional method to ensure the stability of results when appropriate. Bonferroni correction for multiple comparisons was used when appropriate (brain six metabolites and AQ, *p* < 0.05/6 = 0.008; brain six metabolites and QCAE five scales, *p* < 0.05/30 = 0.0016; brain six metabolites and IRI four scales, p < 0.05/24 = 0.002; brain six metabolites and NEO five scales, *p* < 0.05/30 = 0.0016). Differences were set to be significant when *p*-values were < 0.05. Analyses were conducted with SPSS version 20 (IBM). A Comparison of brain metabolites correlations with psychological measurements between the two groups was conducted using cocor R package (two-tailed, alpha = 0.05, confidence level = 0.95, null value = 0).

## Results

### Brain Metabolites in the Medial Prefrontal Cortex and Amygdala

No significant differences between ASD and non-ASD subjects in the contents of glutamate, Glx, NAA, GPC + PC, Cr + PCr, or myo-inositol were found in the medial prefrontal cortex and amygdala (Table 2). Also, glutamine levels in the medial prefrontal cortex and amygdala showed no differences between ASD and non-ASD subjects, although we failed to obtain data from all the participants (Supplementary Table S2). Further, we obtained no statistical significance in the levels of MM09 and MM20 in the medial prefrontal cortex and amygdala in adults with ASD compared with non-ASD controls (Supplementary Table S2).

**Table 2:** Levels of amino acids in the medial prefrontal cortex and amygdala

	Non-ASD control (n = 24)	ASD (n = 24)	*p-*values	*t-*values (df)	Effect size
*Medial prefrontal cortex*					
Glutamate	10.37 ± 2.39	9.63 ± 2.03	0.256	1.15 (46)	0.333
Glx	15.02 ± 2.24	14.76 ± 2.81	0.723	0.35 (46)	0.118
NAA	8.79 ± 1.08	8.85 ± 1.25	0.846	0.17 (46)	0.051
GPC + PC	2.19 ± 0.27	2.30 ± 0.26	0.171	1.44 (46)	0.415
Cr + PCr	7.80 ± 0.71	7.94 ± 0.96	0.570	0.57 (46)	0.165
Myo-inositol	5.77 ± 0.74	5.91 ± 0.91	0.562	0.58 (46)	0.168
*Amygdala*					
Glutamate	7.64 ± 1.39	7.62 ± 1.97	0.974	0.04 (46)	0.011
Glx	10.90 ± 2.25	11.03 ± 3.21	0.874	0.16 (46)	0.046
NAA	7.68 ± 1.32	7.87 ± 1.52	0.641	0.46 (46)	0.133
GPC + PC	2.41 ± 0.49	2.54 ± 0.55	0.394	0.86 (46)	0.249
Cr + PCr	6.69 ± 1.17	7.23 ± 1.69	0.194	1.28 (46)	0.371
Myo-inositol	5.52 ± 1.18	6.15 ± 1.44	0.109	1.65 (46)	0.476

Data are mean ± SD. Effect size represents a sample-based estimate of the quality

*Cr* + *PCr*, Creatine plus phosphocreatine; *Glx*, glutamate plus glutamine; *GPC* + *PC*, glycerophosphorylcholine plus phosphorylcholine; NAA, N-acetyl-L-aspartate

### Associations of Brain Metabolites with Autistic Traits in the ASD Subjects

Among the ASD cohorts, AQ scores failed to show significant relationships with any metabolites in the medial prefrontal cortex (Table 3), but were significantly related with glutamate and Glx contents in the amygdala (r = 0.426, *p* = 0.038; r = 0.407, *p* = 0.048, respectively) (Table 4, Fig. 4). Regression analysis treating BDI, full IQ, verbal IQ and performance IQ as covariates showed no effects of these covariates on the coefficients. Nonparametric coefficients confirmed the correlation between AQ and glutamate in the amygdala of ASD. However, when Bonferroni corrections were done for these results, correlations were not statistically significant (*p* < 0.05/6 = 0.008).

**Table 3:** Relationships among autistic traits, empathy, personality and brain metabolites in the medial prefrontal cortex of adult ASD

ASD (n = 24)	mPF Glutamate	mPF Glx	mPF NAA	mPF GPC + PC	mPF Cr + PCr	mPF Myo-inositol
< Autistic traits >						
AQ	− .195	− .152 b	.219	.074	− .096 #	.154
< Empathy >						
QCAE Perspective-taking	.275	.278	.040	.100	.120	.096
QCAE Online simulation	− .017	− .128	− .124	− .476 *	− .072	− .045
QCAE Emotion contagion	.120	.233	.096	.011	.387 a	.248
QCAE Proximal responsivity	− .033	.102	− .014	− .134	.239	.346
QCAE Peripheral responsivity	− .079	− .164	− .257	− .465 *	− .363	− .388 a
QCAE Cognitive empathy	.149	.094	− .048	− .208	.031	.028
QCAE Affective empathy	.019	.101	− .058	− .238	.160	.119
IRI Perspective-taking	.132	− .047	− .238	− .311	− .016	.171
IRI Empathic concern	.598 ** #	.601 **	.101	.040	.431 *	.218
IRI Personal distress	− .201	.156	.092	.121	.237	.171
IRI Fantasy	− .056	.041	− .378 a	− .084	− .147	− .002
< Personality >						
NEO Neuroticism	− .136	.152	− .157	.099	.048	− .154
NEO Extraversion	.014	− .132	− .317	− .582 **	.046	− .101
NEO Openness	− .044	− .018	− .291	− .424 *	− .111	− .117
NEO Agreeableness	.228	.198	.145	.179	.336	.223
NEO Conscientiousness	− .176	− .181	− .310	− .169	− .209	.154

*p < 0.05, **p < 0 .01, coefficient in the ASD group

^a^*p* < 0.07, a trend for change without significance

^#^ *p* < 0.05, compared to non-ASD group by cocor

^b^*p* < 0.07, a trend for change without significance by cocor

*Glx* glutamate plus glutamine; *NAA* N-acetyl-L-aspartate; *GPC + PC* glycerophosphorylcholine plus phosphorylcholine; *Cr +PCr* Creatine plus phosphocreatine; *AQ* Autism-spectrum Quotient; *IRI* Interpersonal Reactivity Index; NEO, NEO Personality Inventory-Revised; *QCAE* Questionnaire of Cognitive and Affective Empathy; *mPF* medial prefrontal cortex

**Table 4:** Relationships among autistic traits, empathy, personality and brain metabolites in the amygdala of adult ASD

ASD (n = 24)	AMY Glutamate	AMY Glx	AMY NAA	AMY GPC + PC	AMY Cr + PCr	AMY Myo-inositol
< Autistic traits >						
AQ	.426 *	.407 * b	.366	.155	.043	.124
< Empathy >						
QCAE Perspective-taking	− .179	− .144	− .119	.090	.118	.244
QCAE Online simulation	− .295	− .188	− .098	.048	.208 #	.023
QCAE Emotion contagion	− .029	− .093	− .026	.156	.164	.161
QCAE Proximal responsivity	− .147	− .140	.052	.274 ##	.327 #	.110
QCAE Peripheral responsivity	.028	.003	-.080	− .073	− .111	− .226
QCAE Cognitive empathy	− .267	− .188	− .122	.074	.182	.144
QCAE Affective empathy	− .065	− .106	− .025	.167 #	.178 #	.039
IRI Perspective-taking	− .268	− .140	− .170	.102	.196	.108
IRI Empathic concern	.085	− .083	.020	.195	.102	.414 *
IRI Personal distress	.233	.132	.301	.298	.325	.328
IRI Fantasy	.061	.042	− .143	− .060	− .173	− .129
< Personality >						
NEO Neuroticism	− .028	− .178	− .252	− .236	− .297	−.134
NEO Extraversion	− .354	− .373	− .160	.039	.071	− .232
NEO Openness	.157	.230	.104	.246	.249	.122
NEO Agreeableness	− .080	− .126	.060 #	.277	.360 ##	.571 ** ###
NEO Conscientiousness	− .245	− .177	− .071	− .024	− .009	− .189

*p < 0.05, **p < 0 .01, coefficient in the ASD group

^#^*p* < 0.05, ##*p* < 0.01, ###*p* < 0.001, compared to non-ASD group by cocor. ^b^*p* < 0.07, a trend for change without significance by cocor

*Glx* glutamate plus glutamine; *NAA* N-acetyl-L-aspartate; *GPC + PC* glycerophosphorylcholine plus phosphorylcholine; *Cr + PCr* Creatine plus phosphocreatine; *AQ* autism-spectrum Quotient; *IRI* interpersonal reactivity index; NEO, NEO Personality Inventory-Revised; *QCAE* questionnaire of cognitive and affective empathy; *AMY* amygdala

### Associations of Brain Metabolites with Autistic Traits in the Non-ASD Controls

In the non-ASD controls, AQ scores were significantly related with Glx (r = 0.415, *p* = 0.045) and Cr + PCr (r = 0.558, *p* = 0.005) in the medial prefrontal cortex (Table 5), but failed to show significant relationships with any metabolites in the amygdala (Table 6). Regression analysis treating BDI, full IQ, verbal IQ and performance IQ as covariates showed no effects of these covariates on the coefficients. Nonparametric coefficients confirmed the correlation of AQ with Glx and Cr + PCr in the medial prefrontal cortex of non-ASD. When Bonferroni corrections were done for these results, the correlation of AQ with Cr + PCr in the medial prefrontal cortex of non-ASD was statistically significant (*p* < 0.05/6 = 0.008).

**Table 5:** Relationships among autistic traits, empathy, personality and brain metabolites in the medial prefrontal cortex of non-ASD control

Non-ASD control (n = 24)	mPF Glutamate	mPF Glx	mPF NAA	mPF GPC + PC	mPF Cr + PCr	mPF Myo-inositol
< Autistic traits >						
AQ	.319	.414 * b	-.041	− .016	.558** #	.078
< Empathy >						
QCAE Perspective-taking	− .017	− .171	.053	.006	− .301	− .072
QCAE Online simulation	.107	− .045	− .005	− .226	− .313	− .150
QCAE Emotion contagion	− .056	.049	− .014	− .249	.149	.054
QCAE Proximal responsivity	− .241	− .085	− .088	− .088	− .093	.048
QCAE Peripheral responsivity	− .335	− .124	− .409 *	− .224	− .252	− .127
QCAE Cognitive empathy	.035	− .132	.031	− .104	− .333	− .115
QCAE Affective empathy	− .255	− 063	− .202	− .229	− .074	− .006
IRI Perspective-taking	.366	.075	.202	.273	.009	.027
IRI Empathic concern	− .088 #	.167	− .174	− .054	− .028	− .360
IRI Personal distress	.325	.325	.021	.021	.198	− .091
IRI Fantasy	.039	.329	− .152	− .022	.071	− .100
< Personality >						
NEO Neuroticism	.304	.514 *	− .262	.079	.371	− .105
NEO Extraversion	− .311	− .209	− .378 a	− .173	− .423 *	− .094
NEO Openness	.126	.089	− .279	− .260	.219	.001
NEO Agreeableness	.499 *	.294	.208	− .147	− .056	.110
NEO Conscientiousness	− .352	− .438 *	− .040	− .265	− .505 *	− .063

*p < 0.05, **p < 0.01, coefficient in non-ASD subjects

^a^*p* < 0.07, a trend for change without significance

^#^ *p* < 0.05, compared to ASD group by cocor

^b^*p* < 0.07, a trend for change without significance by cocor

*Cr* + *PCr* Creatine plus phosphocreatine; *Glx* glutamate plus glutamine; *GPC* + *PC* glycerophosphorylcholine plus phosphorylcholine; *NAA* N-acetyl-L-aspartate; *AQ* autism-spectrum quotient; *IRI*, interpersonal reactivity index; NEO, NEO Personality Inventory-Revised; *QCAE* Questionnaire of Cognitive and Affective Empathy; *mPF* medial prefrontal cortex

**Table 6:** Relationships among autistic traits, empathy, personality and brain metabolites in the amygdala of non-ASD control

Non-ASD control (n = 24)	AMY Glutamate	AMY Glx	AMY NAA	AMY GPC + PC	AMY Cr + PCr	AMY Myo-inositol
< Autistic traits >						
AQ	− .061	− .141 b	− .077	− .058	.150	.177
< Empathy >						
QCAE Perspective-taking	− .060	.098	− .076	.103	− .237	− .089
QCAE Online simulation	− .252	− .098	− .340	− .157	− .433 * #	− .325
QCAE Emotion contagion	− .335	− .273	− .136	− .417 *	− .390 a	− .193
QCAE Proximal responsivity	− .578 **	− .540 **	− .229	− .492 *	− .494 *	− .331
QCAE Peripheral responsivity	− .234	− .155	.004	− .154	− .195	− .047
QCAE Cognitive empathy	− .159	.002	− .212	− .019	− .357	− .220
QCAE Affective empathy	− .476 *	− .403 a	− .152	− .443 *	− .448 * #	− .239
IRI Perspective-taking	.138	.319	.062	.069	− .110	− .035
IRI Empathic concern	− .195	− .065	− .057	− .146	− .115	− .003
IRI Personal distress	.035	− .091	− .054	− .327	− .203	− .085
IRI Fantasy	− .331	− .187	− .423 *	− .436 *	− .481 *	− .278
< Personality >						
NEO Neuroticism	− .329	− .213	− .322	− .263	− .251	.007
NEO Extraversion	− .233	− .151	− .098	.015	− .131	− .071
NEO Openness	− .485 *	− .461 *	− .282	− .388 a	− .345	− .220
NEO Agreeableness	− .265	− .354	− .519 ** #	− .346	− .455 * ##	− .402 a ###
NEO Conscientiousness	− .033	.044	.034	.098	− .029	− .070

*p < 0.05, **p < 0.01, coefficient in non-ASD subjects

^a^*p* < 0.07, a trend for change without significance

^#^ *p* < 0.05, ## *p* < 0.01, ### *p* < 0.001, compared to non-ASD group by cocor

^b^*p* < 0.07, a trend for change without significance by cocor

*Cr* + *PCr* creatine plus phosphocreatine; *Glx* glutamate plus glutamine; *GPC* + *PC* glycerophosphorylcholine plus phosphorylcholine; NAA, N-acetyl-L-aspartate; *AQ* autism-spectrum Quotient; *IRI* Interpersonal Reactivity Index; NEO, NEO Personality Inventory-Revised; *QCAE* Questionnaire of Cognitive and Affective Empathy; *AMY* amygdala

### Associations of Empathy Subscales on the QCAE and IRI with Brain Metabolites in the ASD Subjects

**Fig. 3:**
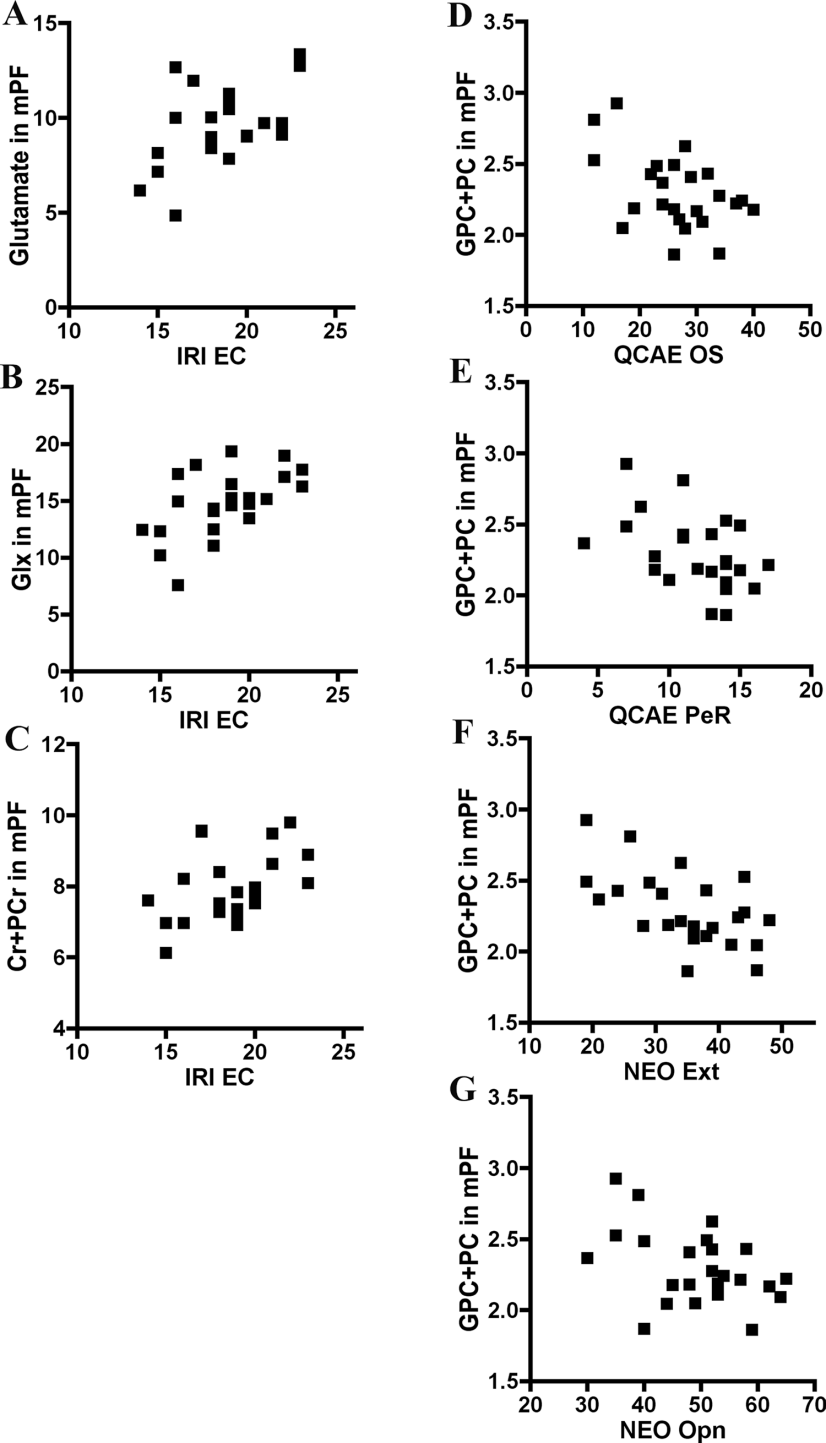
Correlation plots for the association between metabolites in the medial prefrontal cortex and empathy subscales and personality traits in ASD adults. **A**, **B**, **C**: Correlations between empathic concern on the IRI and glutamate, Glx and Cr + PCr in the medial prefrontal cortex of ASD subjects. **D**, **E**: Correlations between online simulation and peripheral responsivity on the QCAE and GPC + PC in the medial prefrontal cortex of ASD subjects. **F**, **G**: Correlations between extraversion and openness on the NEO in the medial prefrontal cortex of ASD subjects. IRI, Interpersonal Reactivity Index; NEO, NEO Personality Inventory-Revised; QCAE, Questionnaire of Cognitive and Affective Empathy; EC, empathic concern; OS, online simulation; PeR, peripheral responsivity; Ext, extraversion; Opn, openness; mPF, medial prefrontal cortex

Online simulation and peripheral responsivity on the QCAE showed significant relationships with GPC + PC (r = -0.476, *p* = 0.019; r = -0.465, *p* = 0.022, respectively) in the medial prefrontal cortex of ASD (Table 3, Fig. 3). Regression analysis treating BDI, full IQ, verbal IQ and performance IQ as covariates showed no effects of these covariates on the coefficients. Nonparametric coefficients confirmed the correlation of peripheral responsivity, but not online simulation, on the QCAE with GPC + PC in the medial prefrontal cortex of ASD. However, when Bonferroni corrections were done for these results, correlations were not statistically significant (*p* < 0.05/30 = 0.0016).

**Fig. 4:**
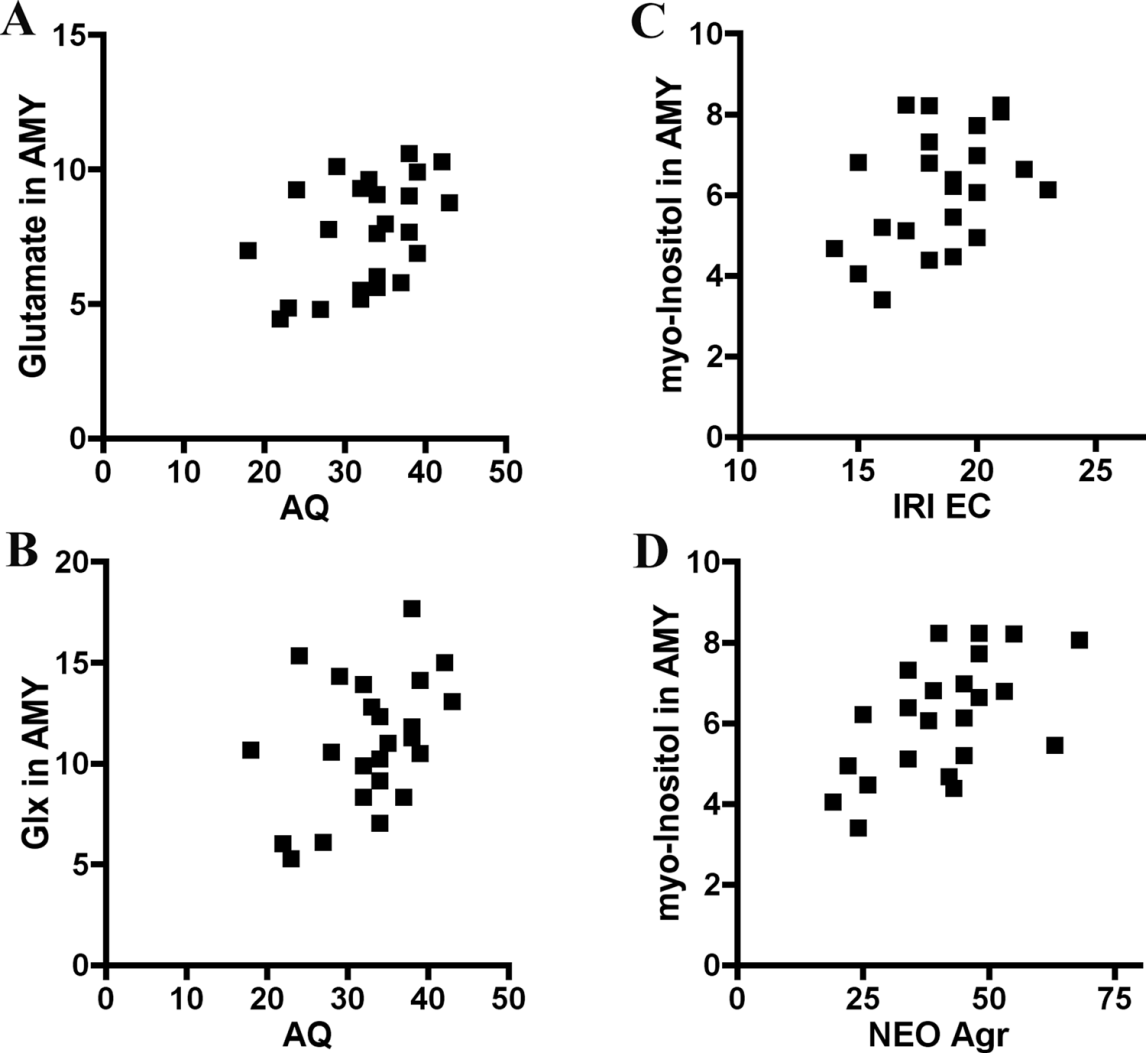
Correlation plots for the association between metabolites in the amygdala and autistic traits, empathy subscales and personality traits in ASD adults. **A**, **B**: Correlations between autistic traits on the AQ and glutamate and Glx in the amygdala of ASD subjects. **C**: Correlations between empathic concern on the IRI and myo-inositol in the amygdala of ASD subjects. **D**: Correlations between agreeableness on the NEO and myo-inositol in the amygdala of ASD subjects. AQ, Autism-Spectrum Quotient; IRI, Interpersonal Reactivity Index; NEO, NEO Personality Inventory-Revised; EC, empathic concern; Agr, agreeableness; AMY, amygdala

Empathic concern on the IRI had significant relationships with glutamate (r = 0.598, *p* = 0.002), Glx (r = 0.601, *p* = 0.002) and Cr + PCr (r = 0.431, *p* = 0.036) in the medial prefrontal cortex in the ASD subjects. (Table 3, Fig. 3). Regression analysis treating BDI, full IQ, verbal IQ and performance IQ as covariates showed no effects of these covariates on the coefficients. Among them, nonparametric coefficients confirmed the correlations of empathic concern on the IRI with glutamate, Glx, and Cr + PCr in the medial prefrontal cortex of ASD. When Bonferroni corrections were done for these results, the correlations of empathic concern on the IRI with glutamate and Glx in the medial prefrontal cortex of the ASD subjects were statistically significant (*p* < 0.05/24 = 0.002). On the contrary, empathic concern on the IRI had significant relationships with myo-inositol in the amygdala in the ASD subjects (r = 0.414, p = 0.050) (Table 4, Fig. 4). Regression analysis treating BDI, full IQ, verbal IQ and performance IQ as covariates showed significant effects of BDI on the coefficients. Nonparametric coefficients did not confirm the correlation.

Perspective taking on the QCAE and IRI did not show significant correlations with any metabolites in the medial prefrontal cortex and amygdala of ASD participants.

### Associations of Personality Traits on the NEO with Brain Metabolites in the ASD Adults

Extraversion and openness on the NEO showed significant correlations with GPC + PC (r = −0.582, *p* = 0.003; r = −0.424, *p* = 0.039, respectively) in the medial prefrontal cortex of ASD (Table 3, Fig. 3). Agreeableness on the NEO showed a significant correlation with myo-inositol (r = 0.571, *p* = 0.003) in the amygdala of ASD (Table 4, Fig. 4). Regression analysis treating BDI, full IQ, verbal IQ and performance IQ as covariates showed no effects of these covariates on the coefficients. Among them, nonparametric coefficients confirmed the correlation of extraversion, but not openness, on the NEO with glutamate in the medial prefrontal cortex of ASD, and that of agreement on the NEO with myo-inositol in the amygdala of ASD. However, when Bonferroni corrections were done for these results, correlations were not statistically significant (*p* < 0.05/30 = 0.0016).

Neuroticism on the NEO failed to show significant correlations with any metabolites in the medial prefrontal cortex and amygdala of the ASD group.

### Correlations of Empathy Subscales on the QCAE and IRI with Brain Metabolites in the Non-ASD Controls

Online simulation on the QCAE significantly correlated with Cr + PCr in the amygdala (r = − 0.433, *p* = 0.034) of non-ASD controls (Table 6). Emotion contagion on the QCAE had a significant correlation with GPC + PC in the amygdala (r = − 0.417, *p* = 0.043) of non-ASD controls (Table 6). Proximal responsivity on the QCAE showed significant correlations with glutamate (r = − 0.578, *p* = 0.003), Glx (r = − 0.540, *p* = 0.006), GPC + PC (r = − 0.492, *p* = 0.015) and Cr + PCr (r = − 0.494, *p* = 0.014) in the amygdala of non-ASD controls (Table 6). Peripheral responsivity on the QCAE had a significant correlation with NAA contents (r = − 0.409, *p* = 0.047) in the medial prefrontal cortex of non-ASD controls (Table 6). Affective empathy on the QCAE showed significant correlations with glutamate (r = − 0.476, *p* = 0.019), GPC + PC (r = − 0.443, *p* = 0.030), and Cr + PCr (r = − 0.448, *p* = 0.028) in the amygdala of non-ASD controls (Table 6). Regression analysis treating BDI, full IQ, verbal IQ and performance IQ as covariates showed no effects of these covariates on the coefficients. Among them, nonparametric coefficients confirmed the correlations of proximal responsivity on the QCAE with NAA in the medial prefrontal cortex of non-ASD, those of peripheral responsivity on the QCAE with glutamate, Glx, GPC + PC, and Cr + PCr in the amygdala of non-ASD and those of affective empathy on the QCAE with glutamate and GPC + PC in the amygdala of non-ASD. However, when Bonferroni corrections were done for these results, correlations were not statistically significant (*p* < 0.05/30 = 0.0016).

Fantasy on the IRI showed significant correlations with NAA (r = − 0.423, *p* = 0.039), GPC + PC (r = − 0.436, *p* = 0.033), Cr + PCr (r = − 0.481, *p* = 0.017) in the amygdala of non-ASD controls (Table 6). Regression analysis treating BDI, full IQ, verbal IQ and performance IQ as covariates showed no effects of these covariates on the coefficients. Among them, nonparametric coefficients confirmed the correlation of Cr + PCr in the amygdala of non-ASD controls. However, when Bonferroni corrections were done for these results, correlations were not statistically significant (*p* < 0.05/30 = 0.0016).

Perspective taking on the QCAE and IRI did not show significant correlations with any metabolites in the medial prefrontal cortex and amygdala of non-ASD control.

### Correlations of Personality Traits on the NEO with Brain Metabolites in the Non-ASD Controls

Neuroticism on the NEO showed a significant correlation with Glx (r = 0.514, *p* = 0.010) in the medial prefrontal cortex of non-ASD controls (Table 6). Extraversion on the NEO showed a significant correlation with Cr + PCr (r = − 0.423, *p* = 0.039) in the medial prefrontal cortex of non-ASD subjects (Table 6). Openness on the NEO showed significant correlations with glutamate (r = − 0.485, *p* = 0.016) and Glx (r = − 0.461, *p* = 0.023) in the amygdala of non-ASD controls (Table 6). Agreeableness on the NEO showed significant correlations with glutamate in the medial prefrontal cortex (r = 0.499, *p* = 0.013), and with NAA and Cr + PCr in the amygdala of non-ASD controls (r = − 0.519, *p* = 0.009; r = − 0.455, *p* = 0.025, respectively) (Table 6). Regression analysis treating BDI, full IQ, verbal IQ and performance IQ as covariates showed no effects of these covariates on the coefficients. Among them, nonparametric coefficients confirmed the correlations of neuroticism with Glx in the medial prefrontal cortex of non-ASD, those of extraversion with Cr + PCr in the medial prefrontal cortex of non-ASD, those of openness with glutamate in the amygdala of non-ASD, and those of agreeableness with NAA and Cr + PCr in the amygdala of non-ASD. However, when Bonferroni corrections were done for these results, correlations were not statistically significant (*p* < 0.05/30 = 0.0016).

By the way, conscientiousness on the NEO showed significant correlations with Glx (r = − 0.438, *p* = 0.032) and Cr + PCr (r = − 0.505, *p* = 0.012) in the medial prefrontal cortex of non-ASD controls (Table 6). Regression analysis treating BDI, full IQ, verbal IQ and performance IQ as covariates showed significant effects of BDI on the coefficients. Nonparametric coefficients confirmed the correlations of conscientiousness with Glx and Cr + PCr in the in the amygdala in the non-ASD controls. However, when Bonferroni corrections were done for these results, correlations were not statistically significant (*p* < 0.05/30 = 0.0016).

### Comparison of Correlations of Brain Metabolites with Psychological Measurements Between ASD and Non-ASD Groups by Cocor

Comparing correlations by cocor between the two groups demonstrated that there existed trends for significance in the correlations of AQ scores with Cr + PCr in the medial prefrontal cortex (z = 1.923, *p* = 0.054, Tables 3 and 5), and with Glx in the amygdala (z = 1.859, *p* = 0.062, Tables 4 and 6).

Comparing correlations by cocor showed significant differences in the correlations of online simulation on the QCAE with Cr + PCr in the amygdala (z = 2.186, *p* = 0.028), in those of proximal responsivity on the QCAE with GPC + PC and Cr + PCr in the amygdala (z = 2.656, *p* = 0.007; z = 2.854, *p* = 0.004, respectively) and in those affective empathy on the QCAE with GPC + PC and Cr + PCr in the amygdala (z = 2.088, *p* = 0.036; z = 2.145, *p* = 0.031, respectively) (hash mark, Tables 4 and 6).

Comparing correlations by cocor showed a significant difference in correlations of empathic concern on the IRI with glutamate in the medial prefrontal cortex (z = 2.521, *p* = 0.011, hash mark, Tables 3 and 5), and significant differences in correlations of agreeableness on the IRI with NAA, Cr + PCr and myo-inositol in the amygdala (z = 2.057, *p* = 0.039; z = 2.812, *p* = 0.004; z = 3.483, *p* = 0.0005, respectively) (hash mark, Tables 4 and 6).

### Associations of Age, Depressiveness on the BDI, and Intelligence on the WAIS-III with Brain Metabolites in the ASD Adults and Non-ASD Controls

We found some significant correlations of age, depressiveness on the BDI, full IQ, verbal IQ and performance IQ with brain metabolites in the medial prefrontal cortex and amygdala in the ASD adults. Detailed information is in the Supplementary Results and Discussion (Supplementary Table S4). Whereas, we did not find significant relationships between brain metabolites in the medial prefrontal cortex and amygdala with age, depressiveness, and IQ in the non-ASD controls (Supplementary Table S5).

## Discussion

The main purpose of this study was to examine the differences in brain metabolite in the brain between ASD adults and non-ASD controls. As shown in Table 7, we found no comprehensive differences in brain metabolites concentrations in the medial prefrontal cortex and amygdala of ASD adults compared with non-ASD controls. No difference in glutamate and Glx levels in the medial prefrontal cortex supports past studies (Murphy et al., 2009; Bernardi et al., 2011; Aoki et al., 2012; Endres et al., 2017; Horder et al., 2018). However, previous studies of ASD adults showed reduced Glx in the medial prefrontal cortex (Bernardi et al., 2011; Tebartz van Elst et al., 2014), and a decrease in glutamate in the anterior cingulate cortex (Tebartz van Elst et al., 2014).

**Table 7:** Summary of MRS data in adult ASD

	Glu	Glx	NAA	GPC + PC	Cr + PCr	Myo-instol
*< Medial prefrontal cortex > *						
Murphy et al, 2002 (1.5 T)	ne	ne	↑	↑	↑	ne
Bernardi et al, 2011 (3 T)	ne	↓	=	=	=	=
Aoki et al, 2012 (3 T)	ne	=	↑	=	=	=
Tebartz van Elst et al, 2014 (3 T)	↓	↓	↓	=	=	=
Endres et al, 2017 (3 T)	ne	=	=	=	↓	↓
Horder et al., 2018 (3 T)	=	=	ne	ne	ne	ne
The present study (3 T)	=	=	=	=	=	=
< Anterior cingulate >						
Libero et al., 2016 (3 T)	ne	=	=	=	=	ne
	Glu	Glx	NAA	GPC + PC	Cr + PCr	Myo-insitol
*< Amygdala >*						
Kleinhans et al, 2009a, 2009b (1.5 T)	ne	ne	=	=	=	=
The present study (3 T)	=	=	=	=	=	=

		Glx/Cr	NAA/Cr	GPC + PC /Cr		
*< Anterior cingulate >*						
Oner et al, 2007 (1.5 T)		ne	↑	=		
Libero et al, 2016 (3 T)		=	↓	=		

*Glx* glutamate plus glutamine; *NAA* N-acetyl-aspartate; *GPC + PC* glycerophosphorylcholine plus phosphorylcholin; *Cr* + *PCr* creatine plus phosphoceatine

^1^H-MRS studies failed to demonstrate consistent results regarding levels of brain metabolites in patients with high functioning ASD or Asperger’s syndrome. The reasons underlying the discrepancies for glutamate and Glx of ASD in the MRS study are currently unclear. This might be due to the subtle distinction in the location of interest: dorsal or ventral medial prefrontal cortex or anterior cingulate cortex. Also, magnetic field strength, scanning methods such as PRESS and STEAM, and particular sequences such as J-resolved point-resolved spectroscopy or J-editing acquisition may be factors for differentiation. Future studies will be needed to address these results.

Next end of this study was to examine the relationships between brain metabolites and psychological features detected by QCAE, IRI and NEO. We found significant correlations of brain metabolites with scores for autistic traits (AQ), empathy (QCAE and IRI) and personality (NEO) among ASD subjects, detected by Pearson correlations. The present study of ASD adults used the same data with our previous study (Shirayama et al., 2022). Significant findings by Pearson parametric correlations were checked for additional nonparametric Spearman's correlation coefficients. Further, Bonferroni correction was done for multiple comparisons when appropriate. Additionally, comparing correlations by cocor were examined to check differences in correlations between the two groups. We think that even though data did not pass Bonferroni correction, statistical differences by cocor will be helpful for future studies. The findings should be taken as preliminary or exploratory. Thus, these data interpretations may be based on effect sizes rather than statistical significance.

Glutamate and Glx in the medial prefrontal cortex in the ASD adults showed significant correlations with empathic concern on the IRI with showed significance, passing Bonferroni corrections thereafter. Furthermore, comparison of correlations by cocor showed that the correlation of glutamate in the medial prefrontal cortex with empathic concern on the IRI in ASD adults was significantly different from that in non-ASD controls. Notably, glutamate in the anterior cingulate was increased in children with ASD (Bejjani et al., 2012) and adolescents with ASD (Joshi et al., 2013). Therefore, it could be that there exists a glutamatergic dysfunction in the medial prefrontal cortex of adults with ASD.

One perspective on the pathophysiology of ASD is the excitation/inhibition imbalance theory, which proposes a relatively high ratio of excitatory to inhibitory neuronal processes (Rubenstein & Merzenich, 2003). In support of this excitation/inhibition imbalance theory, some findings for GABAergic inhibitory deficits have been found in adults with ASD (Coghlan et al., 2012). However, so far, there have been no supporting data for elevated glutamatergic signals in ASD adults. Therefore, the significant correlations of empathic concern on the IRI with glutamate and Glx in the medial prefrontal cortex of ASD adults might support glutamatergic dysfunction in adults with ASD.

Autistic traits on the AQ scores in the ASD adults showed substantially significant relationships with glutamate and Glx in the amygdala. Further, comparing by cocor showed the correlations of correlation of AQ scores with Glx in the amygdala had a trend for significance between the ASD and non-ASD groups. On the contrary, autistic traits on the AQ in the non-ASD showed substantially significant relationships with Glx in the medial prefrontal cortex. Further, comparing by cocor showed that the correlation of AQ scores with Glx in the medial prefrontal cortex had a trend for significance between the ASD and non-ASD groups. The new appearance and lack of correlations in the two groups could play a role in autistic traits on the AQ. Future studies will be needed to address these issues from a specific developing point of view.

Also, Glx in the medial prefrontal cortex of non-ASD exhibited significant correlations with neuroticism and agreeableness on the NEO. However, these correlations of non-ASD did not pass Bonferroni correction, and comparing correlations by cocor showed no differences between two groups. Here, it is noteworthy that neuroticism on the NEO was significantly altered in adults with ASD compared with non-ASD controls. A previous study showed that neuroticism was associated with resting-functional connectivity between the amygdala and medial prefrontal cortex following acute stress (Wang et al., 2018). If this mechanism between stress and neuroticism was conducted via Glx in the medial prefrontal cortex, the lack of correlation in the ASD adult might be related to alterations in neuroticism of ASD adults. Similar things could be said, such as the lack of correlation of agreeableness and openness on the NEO with glutamate and Glx in the in the medial prefrontal cortex of ASD adults, to a lesser extent. Future studies will be needed to elucidate this speculation.

NAA in the amygdala of non-ASD was correlated with agreeableness on the NEO. Although this correlation did not survive Bonferroni corrections, comparing by cocor showed that two correlations of NAA in the amygdala with agreeableness on the NEO were statistically significant between the ASD and non-ASD groups. The lack of correlations in the ASD might make sense to understand the pathophysiology of ASD. NAA is synthesized in the mitochondria of neuron, and catabolized in glia (Rae, 2014). These results could be a key for future studies.

Myo-inositol in the amygdala of ASD adults showed significant relationships with agreeableness on the NEO. Although this correlation did not survived Bonferroni corrections, comparing by cocor showed that the correlation of ASD adults was significantly different from that of non-ASD control. Myo-inositol is involved in phospholipid metabolism as a second messenger in the phophatidylinositol cycle (Kim et al., 2005), and is actively transported into astrocytes, highlighting its role as a biomarker for astrocyte (Barres, 2008). Astrocyte in the amygdala could play a role in the agreeableness of NEO in adults with ASD. via myo-inositol. Future studies will be needed to elucidate this issue.

GPC + PC levels in the medial prefrontal cortex of ASD adults showed significant relationships with online simulation and peripheral responsivity on the QCAE, and with extraversion and openness on the NEO. It is of note that online simulation on the QCAE and extraversion on the NEO were significantly altered in adults with ASD. It is likely that GPC + PC levels in the medial prefrontal cortex of ASD adults are involved in online simulation on the QCAE, and in extraversion on the NEO, However, these correlations did not pass Bonferroni correction, and were not significantly different between the two groups by comparing cocor. GPC + PC reflects membrane phospholipid turnover, and comes from phophatidylcholine and lysophosphatidylcholine via phosphodiesterase including phospholipase C, A2 and lysophospholipase. Recently, phosphodiesterase inhibitors have been proposed to treat ASD (Delhaye & Bardoni, 2021). Therefore, GPC + PC in the medial prefrontal cortex might be a good marker for ASD in future studies.

Cr + PCr in the medial prefrontal cortex of non-ASD showed significant relationships with autistic traits on the AQ, passing the Bonferroni correction thereafter. Further, comparing correlations by cocor showed that the correlation of Cr + PCr in the medial prefrontal cortex with AQ scores in the non-ASD control was statistically different from that of ASD adults. In a typical development, autistic traits on the AQ could be manipulated by Cr + PCr in the medial prefrontal cortex. Conversely, the lack of the correlation of Cr + PCr in the medial prefrontal cortex with AQ scores in ASD adults might be contributed to autistic traits in ASD. PCr is phosphorylated Cr, serving as a reserve of high energy phosphates and converting ADP into ATP where ADP is a major regulator of mitochondrial respiration (Shirayama et al., 2004).

On the other hand, Cr + PCr in the amygdala of non-ASD showed significant correlations with online simulation, peripheral responsivity and affective empathy on the QCAE. Although this correlation did not survive Bonferroni corrections, comparing by cocor showed that the correlations of Cr + PCr in the amygdala with online simulation, peripheral responsivity and affective empathy on the QCAE were statistically significant between the ASD and non-ASD groups. Since online simulation, peripheral responsivity and agreeableness on the NEO were significantly altered in ASD adults, the lack of these correlations might be contributed to alterations in online simulation, peripheral responsivity and agreeableness on the NEO in ASD adults. Future studies will be needed to address these issues from a specific developing point of view.

On the contrary, Cr + PCr in the medial prefrontal cortex of ASD adult showed significant relationships with empathic concern on the IRI. Also, Cr + PCr in the medial prefrontal cortex showed a weak correlation, although not significantly, with emotion contagion on the QCAE. Interestingly, empathic concern on the IRI showed a significant relationship with emotion contagion on the QCAE among ASD adults (Shirayama et al., 2022). However, these correlations did not survive Bonferroni corrections, and the comparison of correlations by cocor did not show statistical significance. Therefore, Cr + PCr in the medial prefrontal cortex might play a role in empathic concern on the IRI and emotion contagion on the QCAE in ASD adults to a lesser extent. Future studies will be needed.

From a psychological point of view, it should be noted that in this study, perspective taking on the QCAE and IRI failed to exhibit any correlations with brain metabolites in the ASD adults and non-ASD controls. It is noteworthy that perspective taking on the QCAE and IRI were significantly altered between the two groups, and perspective taking on the QCAE was a good predictor of autistic traits on the AQ in our recent study (Shirayama et al., 2022). Previous studies reported that perspective taking is associated with the medial prefrontal cortex (D'Argembeau et al., 2007) and that glutamate in the dorsolateral prefrontal cortex, but not the anterior cingulate, correlated perspective taking on the IRI (Montag et al., 2008). It might be that perspective taking of empathy factor is not manipulated by glutamatergic system in the medial prefrontal cortex and amygdala. Future studies will be needed.

This study addressed a gap in the literature about the investigation of brain metabolites by MRS in the medial prefrontal cortex and amygdala of ASD adults and showed substantially or statistically significant associations with autistic traits, empathy, and personality traits. The reasons are as follows: the number of MRS studies of adult ASD was small, the results of past studies were inconsistent, and the number of studies about associations of brain metabolites with autistic traits, empathy, and personality traits in adult ASD was small. A great deal of instability might be acquired using macromolecule removal or additional methods including multiecho techniques.

This study has some limitations. First, sample sizes are small. Second, participants of the two groups showed small differences in IQ between adults with ASD subjects and non-ASD controls despite recruiting participants without intellectual disability (full IQ > 80) (Supplementary Results and Discussion). Third, the ASD group showed a depressive state despite not suffering from depression (Supplementary Results and Discussion). Forth, the large number of comparisons made with clinical phenotype might underestimate the amount of spurious signal that might be present. The findings should be taken as preliminary or exploratory. Finally, there remains a possibility that technical challenges may significantly impact interpretation, which is not clearly articulated nor expressed in sufficient depth because there is difficulty in distinguishing a potentially real relationship from shared methodological variance.

## Conclusion

Adults with ASD showed no differences from non-ASD controls in glutamate, Glx, NAA, GPC + PC, Cr + PCr, or myo-inositol levels in the amygdala or medial prefrontal cortex. However, ASD subjects did show significant correlations of localized brain metabolite levels with autistic traits, empathy components, and personality traits by standard psychological assessments. When Bonferroni corrections were done for these results, the correlations of empathic concern on the IRI with glutamate and Glx in the medial prefrontal cortex of ASD were statistically significant, and the correlation of AQ scores with Cr + PCr in the medial prefrontal cortex of non-ASD was statistically significant.

### Supplementary Information

Below is the link to the electronic supplementary material.

Supplementary Results and Discussion, References (DOCX 18 KB)

Supplementary Table S1 (DOCX 15 KB)

Supplementary Table S2 (DOCX 15 KB)

Supplementary Table S3 (DOCX 15 KB)

Supplementary Table S4 (DOCX 16 KB)

Supplementary Table S5 (DOCX 16 KB)

## References

[CR1] Abell F, Krams M, Ashburner J, Passingham R, Friston K, Frackowiak R, Happé F, Frith C, Frith U (1999). The neuroanatomy of autism: A voxel-based whole brain analysis of structural scans. NeuroReport.

[CR2] Adolphs R (2001). The neurobiology of social cognition. Current Opinion in Neurobiology.

[CR3] Association AP (2013). Diagnostic and statistical manual of mental disorders.

[CR4] Aoki Y, Abe O, Yahata N, Kuwabara H, Natsubori T, Iwashiro N, Takano Y, Inoue H, Kawakubo Y, Gonoi W, Sasaki H, Murakami M, Katsura M, Nippashi Y, Takao H, Kunimatsu A, Matsuzaki H, Tsuchiya KJ, Kato N, Kasai K, Yamasue H (2012). Absence of age-related prefrontal NAA in adults with autism spectrum disorders. Translational Psychiatry.

[CR5] Bachevalier J, Loveland KA (2006). The orbitofrontal-amygdala circuit and self-regulation of social-emotional behavior in autism. Neuroscience and Biobehavioural Reviews.

[CR6] Baron-Cohen S, Ring HA, Bullmore ET, Wheelwright S, Ashwin C, Williams SCR (2000). The amygdala theory of autism. Neuroscience and Biobehavioral Reviews.

[CR7] Baron-Cohen S, Wheelwright S, Skinner R, Martin J, Clubley E (2001). The autism-spectrum quotinent (AQ): Evidence from Asperger syndrome/high-functioning autism, males and females, scientists and mathematicians. Journal of Autism and Developmental Disorders.

[CR8] Barres BA (2008). The mystery and magic of glia: A perspective on their roles in health and disease. Neuron.

[CR9] Beck AT, Ward CH, Mendelson M, Mock J, Erbaugh JK (1961). An inventory for measureing depression. Archives of General Psychiatry.

[CR10] Benjjani A, O'Neill J, Kim JA, Frew AJ, Yee VW, Ly R, Kitchen C, Salamon N, McCracken JT, Toga AW, Alger JR, Levitt JG (2012). Elevated glutamatergic compounds in pregenual anterior cingulate in pediatric autism spectrum disorder demonstrated by ^1^H MRS and ^1^H MRSI. PLoS ONE.

[CR11] Bernardi S, Anagnostou E, Shen J, Kolevzon A, Buxbaum JD, Hollander E, Hof PR, Fan J (2011). In vivo 1H-magnetic resonance spectroscopy study of the attentional networks in autism. Brain Research.

[CR12] Bernhardt BC, Singer T (2012). The neural basis of empathy. Annual Review of Neuroscience.

[CR13] Blair RJ (2008). Fine cuts of empathy and the amygdala: Dissociable deficits in psychopathy and autism. Quaterly Journal Experimental Psychology.

[CR14] Clark JB (1998). N-acetyl aspartate: A marker for neuronal loss or mitochondrial dysfunction. Developmental Neuroscience.

[CR15] Coghlan S, Horder J, Inkster B, Mendez MA, Murphy DG, Nutt DJ (2012). GABA system dysfunction in autism and related disorders: From synapse to symptoms. Neuroscience and Biobehavioral Reviews.

[CR16] Costa PT, McCrae RR (1997). Stability and change in personality assessment: The revised NEO Personality Inventory in the year 2000. Journal of Personality Assessment.

[CR17] Critchley HD, Daly EM, Bullmore ET, Williams SC, Van Amelsvoort T, Robertson DM, Rowe A, Phillips M, McAlonan G, Howlin P, Murphy DG (2000). The functional neuroanatomy of social behavior: Changes in cerebral blood flow when people with autistic disorder process facial expressions. Brain.

[CR18] Dalton KM, Nacewicz BM, Johnstone T, Schaefer HS, Gernsbacher MA, Goldsmith HH, Alexander AL, Davidson RJ (2005). Gaze fixation and the neural circuitry of face processing in autism. Nature Neuroscience.

[CR19] D'Argembeau A, Ruby P, Collette F, Degueldre C, Balteau E, Luxen A, Maquet P, Salmon E (2007). Distinct regions of the medial prefrontal cortex are associated with self-referential processing and perspective taking. Journal of Cognitive Neuroscience.

[CR20] Davis MH (1983). Measuring individual differences in empathy: Evidence for a multidimensional approach. Journal Personality Social Psychology.

[CR21] Delhaye S, Bardoni B (2021). Role of phosphodiesterases in the pathophysiology of neurodevelopmental disorders. Molecular Psychiatry.

[CR22] Endres D, van Elst LT, Meyer SA, Feige B, Nickel K, Bubl A, Riedel A, Ebert D, Lange T, Glauche V, Biscaldi M, Philipsen A, Maier SJ, Perlov E (2017). Gutathione metabolism in the prefrontal brain of adults with high-functioning autism spectrum disorder: An MRS study. Molecular Autism.

[CR23] Eres R, Decety J, Louis WR, Molenberghs P (2015). Individual differences in local grey matter density are associated with differences in affective and cognitive empathy. NeuroImage.

[CR24] Fan J, McCandliss BD, Fossella JI, Fiombanm JI, Posner MI (2005). The activation of attentional networks. Neuroimge.

[CR25] Fan Y, Duncan NW, de Greck M, Northoff G (2011). Is there a core neural network in empathy? An fMRI based quantitative meta-analysis. Neuroscience and Biobehavioural Reviews.

[CR26] Frith CD, Frith U (1999). Interacting minds—a biological basis. Science.

[CR27] Goerlich-Dobre KS, Lamm C, Pripfl J, Habel U, Votinov M (2015). The left amygdala: A shared substrate of alexithymia and empathy. NeuroImage.

[CR28] Haznedar MM, Buchsbaum MS, Wei TC, Hof PR, Cartwright C, Bienstock CA, Hollander E (2000). Limbic circuitry in patients with autism spectrum disorders studies with positron emission tomography and magnetic resonance imaging. American Journal of Psychiatry.

[CR29] Horder J, Petrinovic MM, Mendez MA, Bruns A, Takumi T, Spooren W, Barker GJ, Künnecke B, Murphy DG (2018). Glutamate and GABA in autism spectrum disorder—a translational magnetic resonance spectroscopy study in man and rodent models. Translational Psychiatry.

[CR30] Howard MA, Cowell PE, Boucher J, Broks P, Mayes A, Farrant A, Roberts N (2000). Convergent neuroanatomical and behavioural evidence of an amygdala hypothesis of autism. NeuroReport.

[CR31] Jackson PL, Brunet E, Melzoff AN, Decety J (2006). Empathy examined through the neural mechanisms involved in imagining how I feel versus how you feel pain. Neuropsychologia.

[CR32] Joshi G, Biederman J, Wozniak J, Goldin RL, Crowley D, Furtak S, Lukas SE, Gönenc A (2013). Magnetic resonance spectroscopy study of the glutamatergic system in adolescent males with high-functioning autistic disorder: A pilot study at 4T. European Archives of Psychiatry and Clinical Neuroscience.

[CR33] Jung M, Kosaka H, Saito DN, Ishitobi M, Morita T, Inohara K, Asano M, Arai S, Munesue T, Tomoda A, Wada Y, Sadato N, Okazawa H, Iidaka T (2014). Default mode network in young male adults with autism spectrum disorder: Relationship with autism spectrum traits. Molecular Autism.

[CR34] Kim H, McGrath BM, Silverstone PH (2005). A review of the possible relabvance of inositiol and the phosphatidylinositol second messenger system (PI-cycle) to psychiatric disorders- focus on magnetic resonance spectroscopy (MRS) studies. Human Psychopharmacology.

[CR35] Kleinhans NM, Richards T, Weaver KE, Liang O, Dawson G, Aylward E (2009). Brief report: Biochemical correlates of clinical impairment in high functioning autism and Asperger’s disorder. Journal of Autism and Developmental Disorders.

[CR36] Kleinhans NM, Johnson LC, Richards T, Mahurin R, Greenson J, Dawson G, Aylward E (2009). Reduced neural habituation in the amygdala and social impairments in autism spectrum disorders. American Journal of Psychiatry.

[CR37] Komeda H, Kosaka H, Saito DN, Mano Y, Jung M, Fujii T, Yanaka HT, Munesue T, Ishitobi M, Sato M, Okazawa H (2015). Autistic Empathy toward Autistic Others. Social Cognitive and Neuroscience.

[CR38] Libero LE, Reid MA, White DM, Salibi N, Lahti AC, Kana RK (2016). Biochmistry of the cingulate cortex in autism: An MR spectroscopy study. Autism Research.

[CR39] Lombardo MV, Barnes JL, Wheelwright SJ, Baron-Cohen S (2007). Self-referential cognition and empathy in autism. PLoS ONE.

[CR40] Massaccesi C, Groessing A, Rosenberger LA, Hartmann H, Candini M, di Pellegrino G, Franssinetti F, Silani G (2021). Neural correlates of interpersonal space permeability and flexibility in autism spectrum disorder. Cerebral Cortex.

[CR41] Montag C, Schubert F, Heinz A, Gallinat J (2008). Prefrontal cortex glutamate correlates with mental perspective-taking. PLoS ONE.

[CR42] Mosner MG, McLaurin RE, Kinard JL, Hakimi S, Parelman J, Shah JS, Bizzell J, Green RK, Cernasov PM, Walsh E, Addicott MA, Eisenlohr-Moul T, Carter RM, Dichter GS (2019). Neural mechanisms of reward prediction error in autism spectrum disorder. Autism Research and Treatment.

[CR43] Murphy DGM, Crichley HD, Schmitz N, McAlonan G, van Amelsvoort T, Robertson D, Daly E, Rowe A, Russell A, Simmons A, Murphy KC, Howlin P (2002). Apserger syndrome: A proton magnetic resonance spectroscopy study of brain. Archives of General Psychiatry.

[CR44] Nacewicz BM, Dalton KM, Johnstone T, Long MT, McAuliff EM, Oakes TR, Alexander AL, Davidson RJ (2006). Amygdala volume and nonverbal social impairment in adolescent and adult males with autism. Archives of Geneneral Psychiatry.

[CR45] Nacewicz BM, Angelo L, Dalton KM, Fischer R, Anderle MJ, Alexander AL, Davidson RJ (2012). Reliable non-invasive measurement of human neurochemistry using proton spectroscopy with an anatomically defined amygdala-specific voxel. NeuroImage.

[CR46] Oner O, Devrimci-Ozguven H, Oktem F, Yagmurlu B, Baskak B, Munir KM (2007). Proton MR spectroscopy: Higher right anterior cingulate N-acetylaspartate/choline ratio in Asperger syndrome compared with healthy controls. American Journal of Neuroradiology.

[CR47] Padmanabhan A, Lynch CJ, Schaer M, Menon V (2017). The default mode of network in autism. Biological Psychiatry: Cognitive Neuroscience and Neuroimaging.

[CR48] Pierce K, Müller RA, Ambrose J, Allen G, Courchesne E (2001). Face processing occurs outside the fusiform “face area” in autism: Evidence from functional MRI. Brain.

[CR49] Pretzsch CM, Freyberg J, Voinescu B, Lythgoe D, Horder J, Mendez MA, Wichers R, Ajram L, Ivin G, Haesman M, Edden RA, Williams S, Murphy DGM, Daly E, McAlonan GM (2019). Effects of cannabidiol on brain excitation and inhibition systems: A randomised placebo-controlled single dose trial during magnetic resonance spectroscopy in adults with and without autism spectrum disorder. Neuropsychopharmacology.

[CR50] Provencher SW (2001). Automatic quantification of localized in vivo 1H spectra with LCModel. NMR Biomedicine.

[CR51] Rae CD (2013). A guide to the metabolic pathways and function of metabolites observed in human brain ^1^H magnetic resonance spectra. Neurochemical Research.

[CR52] Rankin KP, Kramer JH, Miller BL (2005). Patterns of cognitive and emotional empathy in frontotemporal lobar degeneration. Cognitive and Behavioral Neurology.

[CR53] Reniers RLEP, Corcoran R, Drake R, Shryane NM, Völlm A (2011). The QCAE: A questionnaire of cognitive and affective empathy. Journal of Personality Assessment.

[CR54] Rivelli A, Mattson MP (2019). Intergenerational metabolic syndrome and neuronal network hyperexcitability in autism. Trends in Neurosciences.

[CR55] Rogers K, Dziobek I, Hassenstab J, Wolf OT, Convit A (2007). Who cares? Revising empathy in Asperger syndrome. Journal of Autism and Developmental Disorders.

[CR56] Rubenstein JL, Merzenich MM (2003). Model of autism: Increased ratio of excitation/inhibition in key neural systems. Genes, Brain and Behavior.

[CR57] Schulkin J (2007). Autism and the amygdala: An endocrine hypothesis. Brain and Cognition.

[CR58] Schurz M, Radua J, Aichhorn M, Richlan F, Perner J (2014). Fractionating theory of mind: A meta-analysis of functional brain imaging studies. Neuroscience and Biobehavioural Reviews.

[CR59] Shirayama Y, Yano T, Takahashi K, Takahashi S, Ogino T (2004). In vivo ^31^P NMR spectroscopy shows an increase in glycerophosphorylcholine concentration without alterations in mitochondrial function in the prefrontal cortex of medicated schizophrenic patients at rest. European Journal of Neuroscience.

[CR60] Shirayama Y, Obata T, Matsuzawa D, Nonaka H, Kanazawa Y, Yoshitome E, Ikehira H, Hashimoto K, Iyo M (2010). Specific metabolites in the medial prefrontal cortex are associated with the neurocognitive deficits in schizophrenia: A preliminary study. NeuroImage.

[CR61] Shirayama Y, Takahashi M, Osone F, Hara A, Okubo T (2017). Myo-inositol, glutamate and glutamine in the prefrontal cortex, hippocampus, and amygdala in major depression. Biological Psychiatry: Cognitive Neuroscience and Neuroimaging.

[CR62] Shirayama Y, Matusmoto K, Hamatani S, Muneoka K, Okada A, Sato K (2022). Associations among autistic traits, cognitive and affective empathy, and personality traits in adults with autism spectrum disorder and no intellectual disability. Scientific Reports.

[CR63] Singer T (2006). The neuronal basis and ontogeny of empathy and mind reading: Review of literature and implications for future research. Neuroscience and Biobehavioural Review.

[CR64] Tebartz van Elst L, Maier S, Fangmeir T, Endres D, Mueller GT, Nickel K, Ebert D, Lange T, Hennig J, Biscaldi M, Riedel A, Perlov E (2014). Disturbed cingulate glutamate metabolism in adults with high-functioning autism disorder: Evidence in support of the excitatoy/inhibitory imbalance hypothesis. Molecular Psychiatry.

[CR65] Veenstra-VanderWeele J, Blakely RD (2012). Networking in autism: Leveraging genetic, biomarker and model system findings in the search for new treatments. Neuropsychopharmacology.

[CR66] Wang Y, Zhu Y, Chen P, Yan F, Chen S, Li G, Hu X, Wang L, Yang Z (2018). Neuroticism is associated with altered resting-state functional connectivity of amygdala following acute stress exposure. Behavioral Brain Research.

[CR100] Watanabe T, Abe O, Kuwabara H, Yahata N, Takano Y, Iwashiro N, Natsubori T, Aoki Y, Takao H, Kawakubo Y, Kamio Y, Kato N, Miyashita Y, Kasai K, Yamasue H (2014). Mitigation of sociocommunicational deficits of autism through oxytocin-induced recovery of medial prefrontal activity: A randomized trial. JAMA Psychiatry.

[CR67] Watanabe T, Yahata N, Abe O, Kuwabara H, Inoue H, Takano Y, Iwashiro N, Natsubori T, Aoki Y, Takao H, Sasaki H, Gonoi W, Murakami M, Katsura M, Kunimatsu A, Kawakubo Y, Matsuzaki H, Tsuchiya K, Kato N, Kano Y, Miyashita Y, Kasai K, Yamasue H (2012). Diminished medial prefrontal activity behind autistic social judgments of incongruent information. PLoS ONE.

[CR68] Wechsler D (2002). WAIS-III, wechsler adult intelligent Scale.

