# Supplementary material for: The Lack of Alterations in Metabolites in the Medial Prefrontal Cortex and Amygdala, but Their Associations with Autistic Traits, Empathy, and Personality Traits in Adults with Autism Spectrum Disorder: A Preliminary Study
Source: J Autism Dev Disord. 2022 Oct 17;54(1):193–210. doi: 10.1007/s10803-022-05778-7 (PMC10791770; doi:10.1007/s10803-022-05778-7)
Supplement: Supplementary file 1 — Supplementary Results and Discussion, References (DOCX 18 KB) [file 10803_2022_5778_MOESM1_ESM.docx]

*Supplementary Results*

*Associations of age, depressiveness on the BDI, and intelligence on the WAIS-III with brain metabolites in the ASD adults*

Age showed a significant correlation with myo-inositol in the medial prefrontal cortex of ASD adults (r = 0.415, *p* = 0.044) (Supplementary Table S4). Regression analysis treating BDI, full IQ, verbal IQ and performance IQ as covariates showed no effects of these covariates on the coefficients. Nonparametric coefficients confirmed the correlations of age with myo-inositol of medial prefrontal cortex in ASD adults (Supplementary Table S4). However, when Bonferroni corrections were done for these results, correlations were not statistically significant (*p* < 0.05/6 = 0.008).

Depressiveness on the BDI showed significant correlations with GPC+PC (r = -0.415, *p* = 0.044), Cr+PCr (r = -0.454, *p* = 0.026), and myo-inositol (r = -0.487, *p* = 0.019) in the amygdala of adult ASD (Supplementary Table S4). Regression analysis treating full IQ, verbal IQ and performance IQ as covariates showed no effects of these covariates on the coefficients. Nonparametric coefficients confirmed the correlations of BDI scores with GPC+PC, Cr+PCr, and myo-inositol of amygdala of adult ASD. However, when Bonferroni corrections were done for these results, correlations were not statistically significant (*p* < 0.05/6 = 0.008). Comparing correlations by cocor between the two groups demonstrated that there existed a trend for significance in the correlations of BDI with myo-inositol in the medial prefrontal cortex (z = 1.815, *p* = 0.069) (Supplementary Tables S4 and S6).

Full IQ showed significant relationships with Cr+PCr (r = 0.423, *p* = 0.039) and myo-inositol (r = 0.503, *p* = 0.012) in the medial prefrontal cortex of adult ASD (Supplementary Table S4). Regression analysis treating BDI as a covariate showed no effects of BDI on the coefficients. Among them, nonparametric coefficients confirmed the correlations of full IQ with myo-inositol of medial prefrontal cortex of adult ASD.

Verbal IQ showed significant relationships with GPC+PC of amygdala of ASD adult (r = 0.408, *p* = 0.048) (Supplementary Table S4). Regression analysis treating BDI and performance IQ as a covariate showed no effects of these covariates on the coefficients. Nonparametric coefficients confirmed the correlations of verbal IQ with GPC+PC of amygdala of adult ASD. However, when Bonferroni corrections were done for these results, correlations were not statistically significant (*p* < 0.05/6 = 0.008).

Performance IQ showed significant relationships with myo-inositol of medial prefrontal cortex (r = 0.618, *p* = 0.001) in ASD adults (Supplementary Table S4). Regression analysis treating BDI and verbal IQ as a covariate showed no effects of BDI and verbal IQ on the coefficients. Nonparametric coefficients confirmed the correlations performance IQ with myo-inositol of medial prefrontal cortex in ASD adults. When Bonferroni corrections were done for these results, the correlations of performance IQ on the WAIS-III with myo-inositol in the medial prefrontal cortex of ASD adults were statistically significant (*p* < 0.05/6 = 0.008). However, Comparing correlations by cocor demonstrated no differences between the two groups.

*Associations of age, depressiveness on the BDI, and intelligence on the WAIS-III with brain metabolites in the non-ASD controls*

We did not find significant relationships between brain metabolites in the medial prefrontal cortex and amygdala with age, depressiveness, and IQ in non-ASD controls (Supplementary Table S5).

*Supplementary Discussion*

In the ASD adults, the correlations of performance IQ with myo-inositol in the medial prefrontal cortex passed Bonferroni corrections. However, the participating ASD adults did not have intellectual disability and a history of delay in language development. Thus, this performance IQ do not reflect general features of adults with ASD adults, but means just characteristics of the participating ASD adults passing the criteria of this study (IQ >80) in the present study. In past studies, we sometimes saw this type of IQ difference in samples. For example, some studies of autism handled IQ as a covariate (Hogeveen et al., 2019, Pretzsch et al., 2019), whereas other studies investigated correlations of empathy with IQ (Komeda et al., 2019, Scheeren et al., 2012). Future study will be needed to elucidate this issue。

As for other characteristics of participants, age showed a significant correlation with myo-inositol in the medial prefrontal cortex of ASD. A previous review study reported age-related frontal NAA levels in autism of children but not of adults (Aoki et al., 2012). The authors suggested that children with ASD had larger brain size and lower NAA levels had early transient brain expansion, which is caused by an increase in non-neuron such as glia cell proliferation. It is likely that the alteration in frontal NAA levels in ASD children disappear in ASD adult following development. Future study will be needed.

BDI scores showed substantially significant relationships with GPC+PC, Cr+PCr, and myo-inositol in the amygdala of ASD. Although these correlations did not survived Bonferroni corrections, comparing by cocor showed that the correlations of myo-inositol with BDI had a trend for significance between the ASD and non-ASD groups. In the correlations of empathic concern with glutamate, Glx and Cr+PCr and those of conscientiousness on the NEO with Glx and Cr+PCr, BDI had significant effects on the coefficients in regression analysis with stepwise method. These correlations might be related with depressive state in autism. However, the participating adults with ASD had never suffered from depression before and at enrollment of the present study. This result reminds us of our previous study that patients with depression, but not the remitted patients, scored a similar pattern of links, high for neuroticism and low for extraversion and conscientiousness on the NEO-PI-R (Takahashi *et al.*, 2013). Thus, it is conceivable that high neuroticism, low extraversion, and low conscientiousness are related to depressive feelings. Indeed, adults with ASD who are considered high functioning display depressive symptoms on the BDI at rates of about 20% to 40% (Lever and Geurts, 2016; Tabartz van Elst *et al.,* 2014; Hollocks *et al.*, 2019). Difficulties in building friendships in case of ASD might lead to unpleasant interpersonal experiences, resulting in social isolation, low self-esteem, and stress. Likewise, treatment resistance might result in burnout and cause depressive states, and suicidal thoughts and behavior are increased among autistic adults compared to the general population (Cassidy *et al.*, 2014). Thus, some character traits might be common to ASD and depression. Future studies will be needed to elucidate the issue.

*Supplementary References*

Cassidy, S., Bradley, P., Robinson, J., Allison, C., McHugh, M. & Baron-Cohen, S. (2014). Suicidal ideation and suicidal plans or attempts in adults with Asperger’s syndrome attending a specialist diagnostic clinic: a clinical cohort study. *Lancet Psychiatry*, 1, 142-7.

Domes, G., Spenthof, I., Radtke, M., Isaksson, A., Normann, C. & Heinrichs, M. (2016). Autistic traits and empathy in chronic vs. episodic depression. *Journal of Affective Disorders*, 195, 144-7.

Hollocks, M. J., Lerh, J. W., Magiati, I., Meister-Stedman, R. & Brugha, T. S. (2019). Anxiety and depression in adults with autism spectrum disorder: a systematic review and meta-analysis. *Psychological Medicine,* 49, 559-72.

Hogeveen, J, Krug, M. K., Geddert, R. M., Ragland, D., & Solomon, M. (2020). Compensatry hippocampal recruitment supports preserved episodic memory in autism spectrum disorder, Biological Psychiatry: Cognitive Neuroscience and Neuroimaging, 5, 97-109.

Komeda, H., Kosaka, H., Fujioka, T., Jung, M., & Okazawa, H. (2019). Do individuals with autism spectrum disorders help other people with autism spectrum disorder? An investigation of empathy and helping motivation in adults with autism spectrum disorder. *Frontiers in Psychiatry*, 10, 376.

Lever, A. G. & Geurts, H. M. (2016). Psychiatric co-occurring symptoms and disorders in young, middle-aged, and older adults with autism spectrum disorder. *Journal of Autism and Developmental Disorders,* 46, 1916-30.

Scheeren, A. M., Koot, H. M., Mundy, P. C., Mous, L., & Begeer, S. (2013). Empathic responsiveness of children and adolescents with high-functioning autism spectrum disorder. *Autism Research*, 6, 363-371.

Takahashi, M., Shirayama, Y., Muneoka, K., Suzuki, M., Sato, K. & Hashimoto, K. (2013). Low openness on the revised NEO personality inventory as a risk factor for treatment-resistant depression. *PLoS ONE,* 8, e71964.
