# Supplementary material for: The Lack of Alterations in Metabolites in the Medial Prefrontal Cortex and Amygdala, but Their Associations with Autistic Traits, Empathy, and Personality Traits in Adults with Autism Spectrum Disorder: A Preliminary Study
Source: J Autism Dev Disord. 2022 Oct 17;54(1):193–210. doi: 10.1007/s10803-022-05778-7 (PMC10791770; doi:10.1007/s10803-022-05778-7)
Supplement: Supplementary file 2 — Supplementary Table S1 (DOCX 15 KB) [file 10803_2022_5778_MOESM2_ESM.docx]

**Supplementary Table S1. The FWHM and SNR in the medial prefrontal cortex and amygdala.**

| Medial prefrontal cortex | Non-ASD control  (n = 24) | ASD  (n = 24) | *p-*values  (uncorrected) |
| --- | --- | --- | --- |
| FWHM (ppm) | 0.066 ± 0.022 | 0.077 ± 0.022 | 0.081 |
| SNR | 14.33 ± 5.17 | 12.79 ± 5.56 | 0.325 |

| Amygdala | Non-ASD control  (n = 24) | ASD  (n = 24) | *p-*values  (uncorrected) |
| --- | --- | --- | --- |
| FWHM (ppm) | 0.086 ± 0.023 | 0.087 ± 0.028 | 0.898 |
| SNR | 20.54 ± 4.68 | 19.54 ± 5.47 | 0.499 |

Data are means ± SD.

FEHM, full-width at half maximum; ppm, parts per million; SNR, signal-to-noise ratio.
