# Supplementary material for: The Lack of Alterations in Metabolites in the Medial Prefrontal Cortex and Amygdala, but Their Associations with Autistic Traits, Empathy, and Personality Traits in Adults with Autism Spectrum Disorder: A Preliminary Study
Source: J Autism Dev Disord. 2022 Oct 17;54(1):193–210. doi: 10.1007/s10803-022-05778-7 (PMC10791770; doi:10.1007/s10803-022-05778-7)
Supplement: Supplementary file 3 — Supplementary Table S2 (DOCX 15 KB) [file 10803_2022_5778_MOESM3_ESM.docx]

**Supplementary Table S2. Concentrations of macromolecules and glutamine in the medial prefrontal cortex and amygdala.**

| Medial prefrontal cortex | Non-ASD control  (n = 24) | ASD  (n = 24) | *p-*values  (uncorrected) |
| --- | --- | --- | --- |
| MM09 | 6.84 ± 1.51  (n = 23) | 6.59 ± 1.38  (n = 24) | 0.571 |
| MM20 | 11.36 ± 3.56  (n = 23) | 10.78 ± 2.37  (n = 21) | 0.529 |
| Glutamine | 5.29 ± 1.61  (n = 21) | 5.26 ± 1.77  (n = 23) | 0.965 |

| Amygdala | Non-ASD control  (n = 24) | ASD  (n = 24) | *p-*values  (uncorrected) |
| --- | --- | --- | --- |
| MM09 | 7.86 ± 1.40  (n = 24) | 7.72 ± 1.24  (n = 24) | 0.714 |
| MM20 | 15.51 ± 2.96  (n = 23) | 14.79 ± 3.00  (n = 24) | 0.412 |
| Glutamine | 3.58 ± 0.99  (n = 20) | 3.88 ± 1.16  (n = 20) | 0.397 |

Data are means ± SD. MM, macromolecule.
