# Supplementary material for: The Lack of Alterations in Metabolites in the Medial Prefrontal Cortex and Amygdala, but Their Associations with Autistic Traits, Empathy, and Personality Traits in Adults with Autism Spectrum Disorder: A Preliminary Study
Source: J Autism Dev Disord. 2022 Oct 17;54(1):193–210. doi: 10.1007/s10803-022-05778-7 (PMC10791770; doi:10.1007/s10803-022-05778-7)
Supplement: Supplementary file 4 — Supplementary Table S3 (DOCX 15 KB) [file 10803_2022_5778_MOESM4_ESM.docx]

**Supplementary Table S3. The voxel compositions of CSF in the medial prefrontal cortex and amygdala.**

| Medial prefrontal cortex | Non-ASD control  (n = 24) | ASD  (n = 24) | *p-*values  (uncorrected) |
| --- | --- | --- | --- |
| CSF | 6.16 ± 2.24 | 6.95 ± 3.46 | 0.349 |

| Amygdala | Non-ASD control  (n = 24) | ASD  (n = 24) | *p-*values  (uncorrected) |
| --- | --- | --- | --- |
| CSF | 4.17 ± 1.56 | 4.82 ± 1.42 | 0.137 |

Deta are means ± SD.

CSF, cerebrospinal fluid.
