# Supplementary material for: The Lack of Alterations in Metabolites in the Medial Prefrontal Cortex and Amygdala, but Their Associations with Autistic Traits, Empathy, and Personality Traits in Adults with Autism Spectrum Disorder: A Preliminary Study
Source: J Autism Dev Disord. 2022 Oct 17;54(1):193–210. doi: 10.1007/s10803-022-05778-7 (PMC10791770; doi:10.1007/s10803-022-05778-7)
Supplement: Supplementary file 5 — Supplementary Table S4 (DOCX 16 KB) [file 10803_2022_5778_MOESM5_ESM.docx]

**Supplementary Table S4. Relationship among age, intelligence, depressive state and brain metabolites in the medial prefrontal cortex and amygdala of ASD adults**

| ASD  (n = 24) | mPF  Glutamate | mPF  Glx | mPF  NAA | mPF  GPC+PC | mPF  Cr+PCr | mPF  Myo-inositol |
| --- | --- | --- | --- | --- | --- | --- |
| Age | -.054 | .030 | -.034 | .007 | .116 | .415 * |
| BDI | -.090 | -.249 | .065 | -.370 | .093 | -.025 |
| FIQ | .114 | .167 | .014 | .423 * | .186 | .503 * |
| VIQ | .036 | .269 | -.016 | .399 a | .304 | .232 |
| PIQ | .174 | -.036 | .048 | .262 | -.047 | .618 ** |

| ASD  (n = 24) | AMY  Glutamate | AMY  Glx | AMY NAA | AMY  GPC+PC | AMY  Cr+PCr | AMY  Myo-inositol |
| --- | --- | --- | --- | --- | --- | --- |
| Age | .193 | .162 | .145 | .345 | .310 | .203 |
| BDI | -.138 | -.088 | -.166 | -.415 * | -.454 * | -.487 * b |
| FIQ | .200 | .304 | .066 | .332 | .207 | .319 |
| VIQ | .234 | .316 | .202 | .408 * | .316 | .315 |
| PIQ | .033 | .112 | -.176 | .055 | -.057 | .179 |

**p* < 0.05, ***p* <0 .01, coefficient in the ASD group. ^a^*p* < 0.07, a trend for change without significance.

^b^*p* <0.07, a trend for change without significance by cocor.

Glx, glutamate plus glutamine; NAA, N-acetyl-L-aspartate; GPC+PC, glycerophosphorylcholine plus phosphorylcholine; Cr+PCr, Creatine plus phosphocreatine; BDI, Beck Depression Inventory; FIQ, Full IQ; VIQ, Verbal IQ; PIQ, Performance IQ; mPF, medial prefrontal cortex; AMY, amygdala.
