# Supplementary material for: The Lack of Alterations in Metabolites in the Medial Prefrontal Cortex and Amygdala, but Their Associations with Autistic Traits, Empathy, and Personality Traits in Adults with Autism Spectrum Disorder: A Preliminary Study
Source: J Autism Dev Disord. 2022 Oct 17;54(1):193–210. doi: 10.1007/s10803-022-05778-7 (PMC10791770; doi:10.1007/s10803-022-05778-7)
Supplement: Supplementary file 6 — Supplementary Table S5 (DOCX 16 KB) [file 10803_2022_5778_MOESM6_ESM.docx]

**Supplementary Table S5. Relationship among age, intelligence, depressive state and brain metabolites in the medial prefrontal cortex and amygdala of non-ASD control.**

| Non-ASD  (n = 24) | mPF  Glutamate | mPF  Glx | mPF  NAA | mPF  GPC+PC | mPF  Cr+PCr | mPF  Myo-inositol |
| --- | --- | --- | --- | --- | --- | --- |
| Age | -.209 | -.147 | -.272 | -.026 | .018 | .133 |
| BDI | .357 | .404 a | -.214 | .052 | .235 | .038 |
| FIQ | .098 | .154 | .265 | -.254 | .187 | .260 |
| VIQ | .086 | .164 | .211 | -.181 | .148 | .214 |
| PIQ | .095 | .083 | .265 | -.292 | .155 | .214 |

| Non-ASD  (n = 24) | AMY  Glutamate | AMY  Glx | AMY NAA | AMY  GPC+PC | AMY  Cr+PCr | AMY  Myo-inositol |
| --- | --- | --- | --- | --- | --- | --- |
| Age | .063 | .056 | .190 | .321 | .338 | .280 |
| BDI | .009 | -.098 | -.247 | -.162 | -.136 | .028 b |
| FIQ | -.015 | -.028 | .175 | .030 | .107 | .032 |
| VIQ | -.018 | -.019 | .147 | .050 | .133 | .031 |
| PIQ | -.034 | -.035 | .168 | .003 | .039 | .004 |

**p* < 0.05, ***p* <0 .01, coefficient in the ASD group. ^a^*p* < 0.07, a trend for change without significance.

^b^*p* <0.07, a trend for change without significance by cocor.

Glx, glutamate plus glutamine; NAA, N-acetyl-L-aspartate; GPC+PC, glycerophosphorylcholine plus phosphorylcholine; Cr+PCr, Creatine plus phosphocreatine; BDI, Beck Depression Inventory; FIQ, Full IQ; VIQ, Verbal IQ; PIQ, Performance IQ; mPF, medial prefrontal cortex; AMY, amygdala.
